# Stress relaxation timescale and hydrogel network connectivity regulate neural progenitor cell stemness and differentiation

**DOI:** 10.1039/d5tb02537k

**Published:** 2026-04-01

**Authors:** Lauren E. Brown, Daphne Bakker, Ping Zhou, Christopher M. Madl

**Affiliations:** a Department of Bioengineering, University of Pennsylvania Philadelphia PA 19104 USA; b Department of Materials Science and Engineering, University of Pennsylvania Philadelphia PA 19104 USA; c Center for Precision Engineering for Health (CPE4H), University of Pennsylvania Philadelphia PA 19104 USA cmadl@seas.upenn.edu

## Abstract

Neural progenitor cells (NPCs) are promising candidates for cell replacement therapies, yet maintaining stemness while enabling expansion in chemically defined three-dimensional (3D) hydrogels remains a challenge. By tuning crosslink exchange kinetics, crosslinker functionality and stoichiometry, polymer phase separation behavior, and adhesive ligand presentation, a family of hydrogels was prepared to study the effects of stress relaxation timescale and network connectivity on NPC phenotype. Hydrogels with rapid relaxation and low connectivity promote expansion of NPCs as distributed single-cell networks that maintain stemness marker expression and differentiation capacity. NPCs embedded in slowly relaxing hydrogels maintained stemness marker expression through cell clustering but exhibited impaired proliferation and differentiation. Similarly, in the absence of integrin-binding cell adhesive ligands, NPCs also maintained stem cell marker expression but remained as clusters rather than distributed single-cell networks. Cadherin cell–cell contacts enable downstream β-catenin signaling and stemness maintenance, which are enhanced in rapidly relaxing, low connectivity networks. These findings identify a combination of network connectivity, stress relaxation timescale, and integrin-binding adhesive ligands as crucial design parameters for maintaining NPC stemness and differentiation capacity in 3D hydrogel networks.

## Abstract 2

A family of modular recombinant protein–synthetic polymer hybrid hydrogels reveals that stress relaxation timescale and network connectivity regulate neural progenitor cell stemmness.

## Graphical abstract

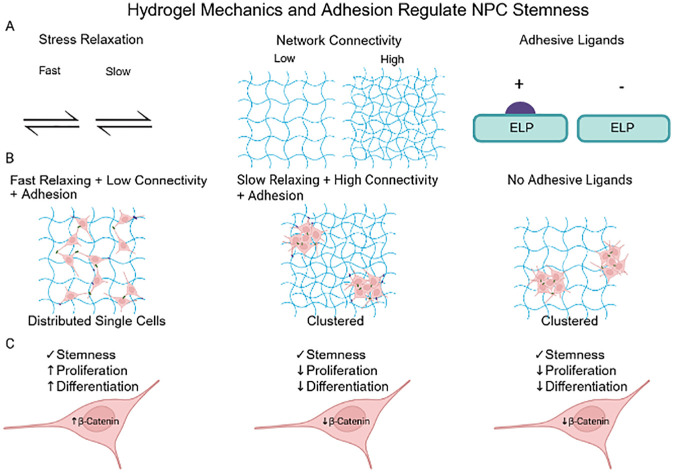

## Introduction

NPCs capable of self-renewal and differentiation into neurons and glia are an attractive cell population for *in vitro* brain modeling and for cell replacement therapies to treat neurodegenerative diseases (NDDs), traumatic brain injury, and spinal cord injury.^1,2^ The progressive loss of neurons exhibited in NDDs and following injury, coupled with the central nervous system's inherent lack of regeneration, affords a great challenge that could be addressed by cell transplantation therapies.^2^ However, strategies for robust expansion of NPCs while maintaining stemness remain limited.^3^

*In vivo*, stem cells reside in a specialized niche, a cell and tissue specific microenvironment consisting of the extracellular matrix (ECM) and various other cell types that together provide key biophysical and biochemical cues regulating stem cell fate.^4^ The ECM is a dynamic and remodelable network of macromolecules that engages in crosstalk with the resident cells.^5–8^ Thus, the development of highly tunable biomaterial platforms that mimic cell–ECM interactions and support NPC maintenance and expansion is a promising approach to enable cell transplantation therapies.^9^

Native brain ECM can be dynamically remodeled by cells through enzymatic degradation. Furthermore, brain tissue is a highly viscoelastic material that can dissipate stress imposed by cellular forces through rearrangement of the ECM. A characteristic feature of viscoelastic materials is their ability to relax applied stresses over defined timescales.^10^ Relaxation timescales are typically reported as the time for the stress in the network to relax to half its initial value (*τ*_1/2_), allowing ease of comparison among different hydrogel compositions. Brain tissue exhibits a *τ*_1/2_ of approximately 10^2^ s.^10–12^ Crucially, this timescale corresponds to the timescales of focal adhesion and cytoskeletal dynamics. In the motor clutch model for cell adhesion, actin polymerization at the cell leading edge is countered by myosin contractility creating actin retrograde flow.^10^ On soft substrates, matching the relaxation timescale to clutch binding and unbinding (*τ*_1/2_ ∼ 10^2^ s) results in maximal cell spreading and migration.^10,13,14^ This timescale also promotes cell spreading within 3D hydrogel matrices.^15^

Previous studies have reported that matrix remodeling through proteolysis or physical reorganization is necessary for proper NPC function.^11,16–18^ Matrix remodeling mediates cell–cell N-cadherin contacts and downstream β-catenin signaling, which regulates stem cell maintenance genes.^16,19^ Prior work revealed that engineering hydrogel networks with high degrees of proteolytic degradability or with rapid stress relaxation timescales on the order of 10^2^ s could support cell–cell contact and stemness maintenance.^16^ A more recent study reported that viscoelastic matrix remodeling could enable stemness maintenance at much slower stress relaxation timescales (*τ*_1/2_ ∼ 10^4^–10^5^ s) but that N-cadherin binding sites were required in addition to integrin-binding sites to enable expansion as single cells. This single-cell phenotype only occurred in the faster relaxing (10^4^ s) materials. In slower relaxing materials, or in materials only presenting integrin-binding ligands, the NPCs instead maintained cell–cell contacts through expansion as cellular clusters.^11^ We hypothesized that rapidly relaxing matrices (*τ*_1/2_ ∼ 10^2^–10^3^ s) with low degrees of network connectivity to enable proteolytic degradation would permit stemness maintenance and differentiation of distributed networks of single NPCs.

Viscoelastic stress relaxation can be engineered into hydrogels through incorporation of dynamic covalent crosslinking chemistries.^10^ We adapted prior systems using proteolytically-degradable elastin-like proteins (ELPs) crosslinked *via* hydrazone bonds to achieve relevant timescales of stress relaxation (*τ*_1/2_ ∼ 10^2^–10^5^ s).^20,21^ Engineered protein materials like ELPs are uniquely advantageous due to their ability to simultaneously regulate stiffness, stress relaxation timescales, degradability, and adhesive ligand presentation.^16,22^ ELPs provide an additional advantage due to their relatively hydrophobic sequences, which undergo thermally-driven phase separation and resist swelling, enhancing the stability of hydrazone-based materials that are subject to hydrolytic degradation.^21,23^

In this study, we systematically tuned crosslink exchange kinetics, hydrogel network connectivity, polymer phase separation behavior, and adhesive ligand presentation to elucidate the effects of stress relaxation timescale and network structure on NPC stemness maintenance. We demonstrate that hydrogels with lower connectivity and faster stress relaxation better support NPC spreading and stemness maintenance. At sufficiently rapid stress relaxation timescales, spread NPCs were able to maintain stemness and differentiation potential, requiring only integrin-mediated adhesion. Hydrogels with high connectivity and slower relaxation timescales only enabled maintenance of stemness marker expression by cell clustering, and the resulting clustered cells had diminished proliferation and differentiation capacity. While altering the phase separation behavior of the ELPs decreased stress relaxation timescales, it did not have a distinct effect on NPC phenotype. Ultimately, our results demonstrate that a combination of network connectivity, stress relaxation, and integrin-binding adhesive ligands mediate NPC stemness maintenance and differentiation.

## Results

**Fig. 1:**
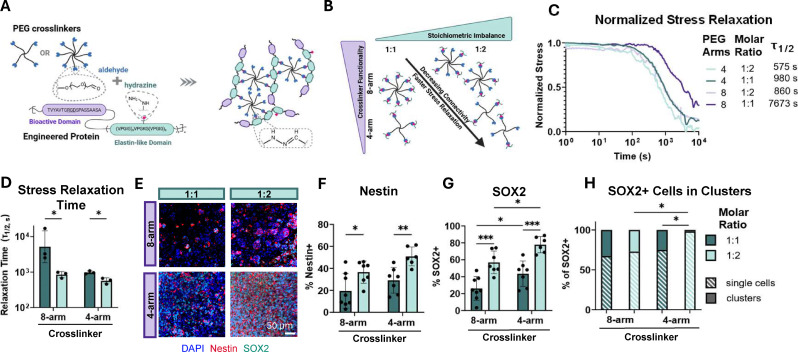
Decreased network connectivity and faster stress relaxation promote NPC maintenance. (A) Schematic of hydrazine-modified elastin-like proteins (ELPs) crosslinked with aliphatic aldehyde modified multi-arm poly(ethylene glycol) (PEG) to create a viscoelastic dynamic covalent hydrogel network. (B) Schematic depicting how crosslinker functionality and reactive group stoichiometry regulate network connectivity and stress relaxation timescales. (C) Normalized representative stress relaxation curves. Stress relaxation tests were completed under a constant strain of 10% at 37 °C. (D) Stress relaxation half-times (*τ*_1/2_) (*n* = 3). (E) Representative maximum projection fluorescence images of NPCs after 7 days of culture stained for neural stem cell markers Nestin (red) and SOX2 (green) and DAPI (blue). Quantification of the percentage of (F) Nestin+ cells and (G) SOX2+ cells after 7 days in culture (*n* = 6–8). (H) Quantification of the percentage of SOX2+ cells that are in clusters *vs.* single cells after 7 days in culture (*n* = 6–8). Statistical analyses performed as lognormal unpaired *t*-test (D), and two-way ANOVA with Bonferroni *post hoc* tests (F)–(H). *p < 0.05, **p < 0.01, ***p < 0.001. Data plotted as mean ± standard deviation. Confidence intervals and *p*-values are reported in Table S1 (F)–(H).

To investigate the effects of hydrogel network properties on NPCs, ELPs were used to generate a set of hydrogels with a range of network connectivity and stress relaxation timescales. The ELPs contain a bioactive domain with a fibronectin-derived arginine–glycine–aspartic acid (RGD) integrin binding site that is recognized by NPCs.^24,25^ The bioactive domain has also been shown to be the predominant site of proteolytic degradation, as elastin-like sequences are relatively resistant to proteolysis.^16,26^ The bioactive domains are flanked by three repeating elastin-like domains that provide selective crosslinking sites.^25,27^ To enable stress relaxation and incorporate dynamic covalent crosslinks into the hydrogels, the lysines in the elastin-like domains were modified with hydrazine moieties.^21^ NMR characterization confirmed complete functionalization of the primary amines with hydrazinoacetic acid (Fig. S1). For rapid relaxation, the hydrazine-ELPs were crosslinked with multi-arm poly(ethylene glycol) (PEG) macromers bearing aliphatic aldehydes with 4-arm or 8-arm molecular architectures (Fig. 1A).^28^ Synthetic multi-arm star PEG crosslinkers were chosen to precisely alter network architecture and restrict degradation to the ELP bioactive site. Prior studies demonstrating 3D culture of NPCs in ELP and hyaluronic acid (HA) containing hydrogels may exhibit confounding effects due to the presence of the HA.^11,12,21^ HA is an inherently bioactive material that engages cell surface receptors, such as CD44, and can be degraded by the cell-secreted enzyme hyaluronidase.^12,20,29^ The use of PEG crosslinkers restricts cell adhesion and degradation primarily to the ELP bioactive site, permitting more carefully controlled mechanistic studies. The ELP–PEG system is highly modular, allowing control over crosslinking architecture, stress relaxation timescale, and degradation. Furthermore, the lower connectivities attainable with the PEG crosslinkers allow the hydrogels to achieve relaxation half-times similar to native brain tissue (∼10^2^ s). The dimensionality of the culture format (2D *vs.* 3D) plays a key role in regulating cell fate. Cells embedded in a 3D matrix experience force in all directions and must rearrange the matrix to spread.^30^ To more closely mimic native brain ECM and its viscoelastic properties, NPCs were embedded within 3D hydrogels. All hydrogels exhibited storage moduli within a physiologically relevant range for brain tissue (Fig. S2) (∼0.1–1 kPa).^31^

We first sought to investigate the effects of network connectivity by varying the stoichiometric balance between hydrazine and aldehyde reactive groups and by regulating the crosslinker functionality (Fig. 1B). A constant polymer concentration and cell-adhesive ligand density was maintained by forming all gels with 3 wt% ELP and using PEG crosslinkers with consistent arm lengths. Utilizing two stoichiometric ratios of 1 : 1 and 1 : 2 of hydrazine to aldehydes respectively, with either 4-arm or 8-arm PEG crosslinkers, afforded hydrogels with a range of connectivity and stress relaxation timescales (Fig. 1C and D). The stress relaxation half-time (*τ*_1/2_), which is the time required to dissipate half of the initial stress under a constant applied strain, ranged from ∼7500 s for 1 : 1 stoichiometrically balanced 8-arm PEG gels to ∼570 s for 1 : 2 stoichiometrically imbalanced 4-arm PEG gels (Fig. 1C and D).

To assess NPC stemness maintenance, single-cell suspensions of NPCs were encapsulated in the hydrogels. Cell viability in culture was greater than 90%, and no difference in viability was observed as a function of aldehyde concentration (Fig. S3). Immunostaining for the classical stemness markers Nestin and SOX2 was performed 7 days after encapsulation and culture in maintenance medium (Fig. 1E). As expected, decreasing stress relaxation timescale by applying a stoichiometric imbalance and lower network connectivity resulted in an increase in Nestin and SOX2 positive NPCs (Fig. 1E–G). Cells embedded in stoichiometrically imbalanced matrices displayed significantly higher levels of Nestin and SOX2 within both 8-arm and 4-arm PEG gels (Fig. 1F and G). Furthermore, decreasing matrix connectivity by using a 4-arm PEG crosslinker afforded an increase in Nestin and SOX2 expression across respective stoichiometric ratios when compared to higher connectivity 8-arm crosslinkers (Fig. 1F and G). As observed in a recent study, at slow stress relaxation timescales, NPCs aggregate to form clusters to maintain stemness (Fig. 1E and H), likely through N-cadherin cell–cell contacts.^11^ Achieving a fast enough stress relaxation timescale significantly increased the amount of SOX2 positive single cells embedded in the hydrogels (Fig. 1H).

It is important to note that altering crosslinker functionality (4-arm *vs.* 8-arm) and stoichiometric ratio changes both the network connectivity and the relaxation timescale. Engineering a stoichiometric imbalance with excess aldehydes further reduces the effective crosslinker functionality (Fig. 1B). As in prior studies investigating the role of network connectivity in ELP–PEG hybrid hydrogels, the molecular weights of the PEG crosslinkers were chosen such that the arm lengths are consistent across conditions, preserving the distance between crosslinks and minimizing differences in crosslink density, stiffness, and mesh size.^22^ However, changing the stoichiometric ratio of crosslinker inherently alters the total polymer content of the hydrogels, which may in turn alter polymer chain mobility and related network properties, highlighting the difficulties in fully decoupling matrix properties in 3D. Taken together, decreasing network connectivity and faster stress relaxation timescales promote NPC stemness maintenance.

**Fig. 2:**
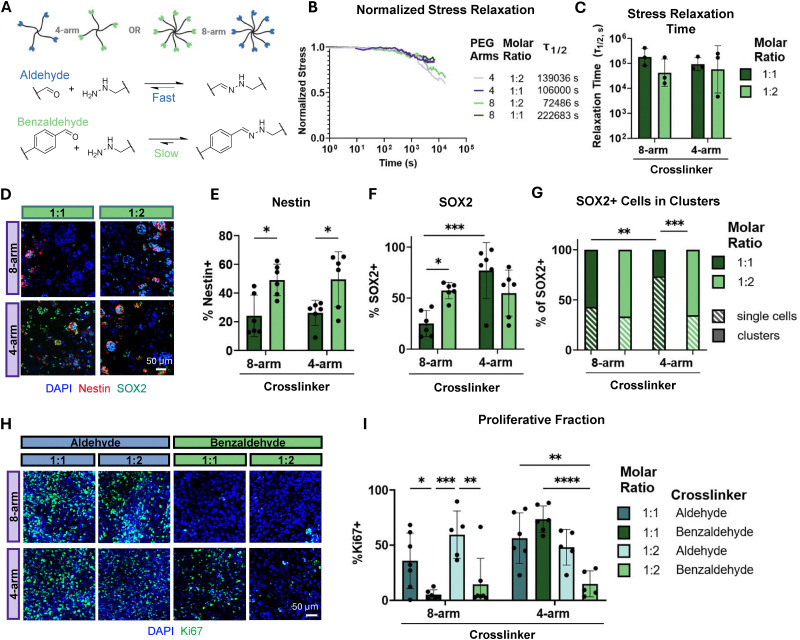
Slow stress relaxing hydrogels impair proliferation and promote cell clustering. (A) Schematic of aliphatic aldehyde or benzaldehyde modified multi-arm PEG crosslinkers. Fast relaxing hydrogels contain only aliphatic aldehyde-modified PEG, and slow relaxing gels contain only benzaldehyde-modified PEG. (B) Normalized representative stress relaxation curves for benzaldehyde crosslinked hydrogels. (C) Stress relaxation half-times (*τ*_1/2_) for benzaldehyde crosslinked gel conditions (*n* = 3). (D) Representative maximum projection fluorescence images of NPCs encapsulated in benzaldehyde crosslinked hydrogels after 7 days of culture stained for neural stem cell markers Nestin (red) and SOX2 (green) and DAPI (blue). Quantification of the percentage of (E) Nestin+ cells and (F) SOX2+ cells after 7 days in culture (*n* = 6). (G) Quantification of the percentage of SOX2+ cells that are in clusters *vs.* single cells after 7 days in culture (*n* = 6). (H) Representative maximum projection fluorescence images of NPCs encapsulated in aliphatic aldehyde or benzaldehyde crosslinked hydrogels after 7 days of culture stained for the proliferation marker Ki67 (green) and DAPI (blue). (I) Quantification of the percentage of Ki67+ cells after 7 days in culture (*n* = 3–4). Statistical analyses performed as lognormal unpaired *t*-test (C), and two-way ANOVA with Bonferroni multiple comparisons test (E)–(G) and (I). *p < 0.05, **p < 0.01, ***p < 0.001 ****p < 0.0001. Data plotted as mean ± standard deviation. Confidence intervals and *p*-values are reported in Tables S1 (E)–(G) and S2 (I).

As network connectivity appeared to be a critical regulator of stemness maintenance in addition to stress relaxation timescale, we next sought to investigate the effect of network connectivity in hydrogels with longer stress relaxation times. Aromatic aldehydes, like benzaldehyde, form hydrazone bonds with slower off rates compared to aliphatic aldehydes.^28^ Varying the ratio between aldehyde and benzaldehyde functional groups in ELP gels can achieve a range of stress relaxation timescales due to the slower benzaldehyde bond exchange kinetics.^11,12^ We chose to expand our family of hydrogels from Fig. 1 by functionalizing PEG molecules with benzaldehydes, rather than aliphatic aldehydes, to achieve a range of long stress relaxation times (∼10^4^–10^5^ s) (Fig. 2A–C). Physiologically relevant storage moduli were maintained for each condition (Fig. S4). Consistent with the aldehyde-functionalized gels, increasing the stoichiometric imbalance and decreasing the crosslinker functionality resulted in faster stress relaxation timescales (Fig. 2B and C). Consistent with the aliphatic aldehyde gels, cell viability in the benzaldehyde gels was greater than 90%, independent of benzaldehyde concentration (Fig. S3). Immunostaining confirmed maintenance of stemness marker expression in NPC clusters, and loss of stemness in isolated single cells (Fig. 2D–G). For each gel composition, less single cells were observed in the benzaldehyde gels compared to the corresponding aliphatic aldehyde gels (Fig. 1H and 2G).

A hallmark of stem cell function is the ability to proliferate and self-renew. To assess differences in proliferation, immunostaining for the common proliferation marker Ki67 was performed in both aliphatic aldehyde and benzaldehyde gels on day 7 (Fig. 2H). The proliferative fraction increased as a function of lower matrix connectivity and faster stress relaxation time (Fig. 2I). Cells embedded in benzaldehyde crosslinked gels largely lost their proliferation capacity, even in lower connectivity networks (Fig. 2I). Cells rearrange their cytoskeleton within seconds of an applied force and spread within minutes. Thus, we reason that benzaldehyde containing gels relax too slowly to allow for cytoskeletal rearrangement, spreading, and subsequent proliferation.^32^ Together, these results demonstrate that carefully engineering network connectivity and stress relaxation into biomaterial platforms is a crucial consideration when designing for NPC stemness maintenance.

**Fig. 3:**
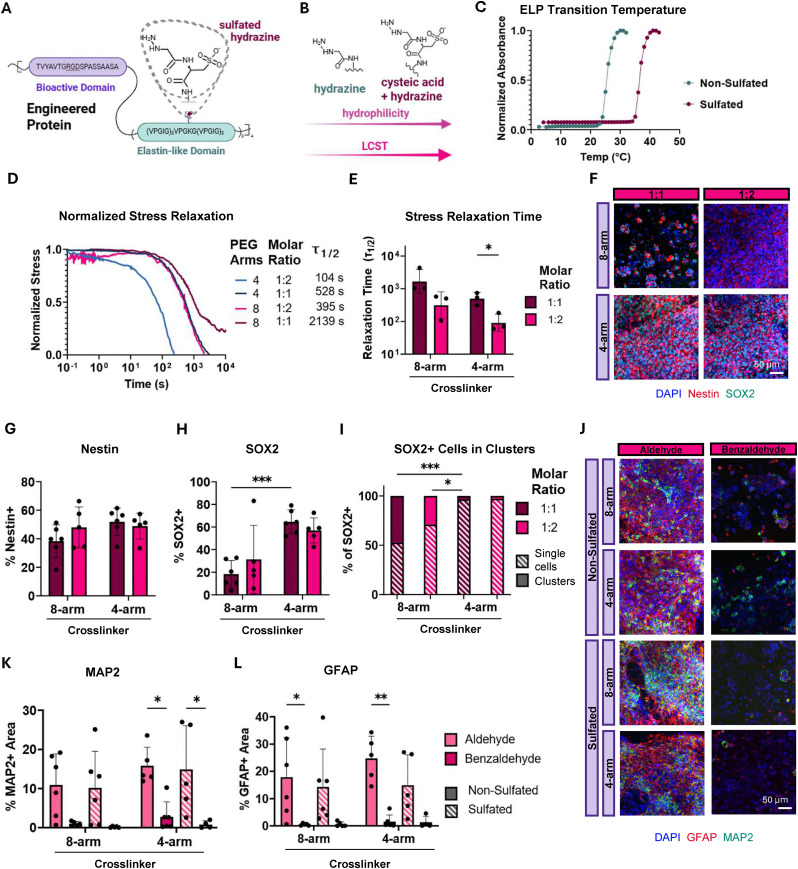
Sulfation increases ELP LCST, decreasing stress relaxation timescale and maintaining NPC stemness. (A) Schematic of sulfated ELP-hydrazine crosslinked with aldehyde-modified PEGs. (B) Schematic depicting the relationship between sulfation, hydrophilicity, and increasing ELP LCST. (C) Representative solution turbidity curves of non-sulfated hydrazine-modified ELP and sulfated hydrazine-modified ELP (1 w/v% in PBS). (D) Normalized representative stress relaxation curves for aliphatic aldehyde crosslinked sulfated-ELP hydrogels. (E) Stress relaxation half-times (*τ*_1/2_) for aliphatic aldehyde crosslinked sulfated-ELP hydrogels (*n* = 3). (F) Representative maximum projection fluorescence images of NPCs encapsulated in aliphatic aldehyde crosslinked sulfated-ELP hydrogels after 7 days of culture stained for neural stem cell markers Nestin (red) and SOX2 (green) and DAPI (blue). Quantification of the percentage of (G) Nestin+ cells and (H) SOX2+ cells after 7 days in culture (*n* = 5–6). (I) Quantification of the percentage of SOX2+ cells that are in clusters *vs.* single cells after 7 days in culture (*n* = 5–6). (J) Representative maximum projection fluorescence images of NPCs encapsulated in aliphatic aldehyde and benzaldehyde crosslinked non-sulfated and sulfated ELP-hydrogels after 14 days of culture stained for the neuronal marker MAP2 (green), the astrocytic marker GFAP (red), and DAPI (blue). Quantification of the fraction of (K) MAP2+ and (L) GFAP+ area after 14 days in culture (*n* = 5–6). Statistical analyses performed as lognormal unpaired *t*-test (E), and two-way ANOVA with Bonferroni multiple comparisons test (G)–(I) and (K), (L). *p < 0.05, **p < 0.01, ***p < 0.001. Data plotted as mean ± standard deviation. Confidence intervals and *p*-values are reported in Tables S1(G)–(I), S3(K) and S4(L).

To further optimize the gel properties, we next investigated phase separation-induced effects on hydrogel architecture. ELPs are intrinsically disordered proteins that remain soluble until heated above a lower critical solution temperature (LCST) which triggers phase separation into an insoluble protein-rich state. During this phase transition, the polymer chains collapse to expose hydrophobic side chains and cluster proline residues inward.^33^ The hydrazine-modified ELPs exhibit an LCST at 25–26 °C, based on solution turbidity measurements (Fig. 3C). To increase the LCST and limit phase separation under cell culture conditions, the ELPs were modified with hydrophilic cysteic acids at the primary amine sites prior to introduction of the hydrazine crosslinking groups (Fig. 3A and B).^28,34^ NMR characterization confirmed complete functionalization with cysteic acid and hydrazinoacetic acid functionalities (Fig. S5). As protein aggregation should slow stress relaxation due to physical entanglement of the polymer chains, we postulated that increasing the LCST would enhance cell spreading, stemness maintenance, and differentiation by decreasing the relaxation time of the hydrogels. As the net effect of the cysteic acid modification is to add negatively charged sulfate groups, the original hydrazine-modified ELP will be referred to as “non-sulfated” and the cysteic-acid and hydrazine modified ELP as “sulfated.” The sulfated ELP exhibits an LCST near cell culture conditions (36–37 °C) (Fig. 3C). In addition to changing the critical temperature for the LCST, sulfation also alters the isoelectric point of the proteins. While screening by counter ions in the media should limit the effects of the low density of additional negative charges, it is important to note that surface charges can also alter cell behavior.

The same PEG crosslinker conditions were used for the sulfated ELPs as for the non-sulfated ELPs in Fig. 1 and 2. All hydrogel compositions maintained a storage modulus within a relevant range for brain tissue (Fig. S6). Decreasing the number of PEG arms while increasing the stoichiometric imbalance in sulfated gels achieved the fastest stress relaxing conditions, with relaxation half-times as low as 10^2^ s, which were faster compared to non-sulfated aliphatic aldehyde crosslinked hydrogels (Fig. 3D, E and Fig. S7). Immunostaining confirmed Nestin and SOX2 positive cells on day 7 in all gel conditions (Fig. 3G–I). Increasing the stoichiometric imbalance and decreasing the PEG crosslinker functionality afforded more spread single cells expressing Nestin and SOX2 (Fig. 3G and F). Cells embedded in sulfated ELP crosslinked with 4-arm PEGs exhibited the highest percentage of SOX2+ single cells and very few cell clusters (Fig. 3F). The trends observed in the non-sulfated ELP crosslinked with PEG-benzaldehyde held true for the sulfated-ELP crosslinked with PEG-benzaldehyde (Fig. S8). Hydrogels prepared from sulfated ELP crosslinked with PEG-benzaldehyde did achieve slightly faster stress relaxation timescales compared to the non-sulfated conditions (Fig. S9). As for the non-sulfated samples, the aliphatic aldehyde crosslinked sulfated ELP gels largely displayed higher proliferative fractions on day 7 compared to benzaldehyde crosslinked sulfated ELP (Fig. S10 and Table S5). While increasing the LCST through sulfation itself does not appear to have a drastic effect on cell morphology, it does enable rapid stress relaxation timescales that approach those of native brain tissue and facilitates stemness maintenance as single cells with only integrin-binding adhesive ligands.

Beyond maintaining stemness marker expression and proliferation capacity, proper NPC function includes the capacity to differentiate into neurons and glial cells such as astrocytes. We hypothesized that, because cells in fast relaxing hydrogels form distributed single-cell networks and exhibit higher proliferative fraction, cells embedded within these hydrogels would exhibit enhanced differentiation compared to cells encapsulated in hydrogels with slower stress relaxation timescales. Cells were cultured for 7 days in standard maintenance medium, which is supplemented with epidermal growth factor (EGF) and fibroblast growth factor-2 (FGF-2), to enable matrix reorganization and then cultured for an additional 7 days in the absence of growth factors to induce differentiation. To assess differentiation, the samples were stained on day 14 for the neuronal marker microtubule-associated protein 2 (MAP2) and the astrocytic marker glial fibrillary acidic protein (GFAP). To best assess the impact of stress relaxation time on NPC differentiation, we embedded cells in a subset of the gels, covering both short and long stress relaxation half-times. Consistent with our hypothesis, immunostaining revealed well-distributed networks of neurons and astrocytes at short stress relaxation half-times and limited differentiation capabilities in cell clusters formed at longer relaxation times (Fig. 3J). NPCs exhibited noticeably higher levels of MAP2 and GFAP expression in aliphatic aldehyde containing gels compared to benzaldehyde counterparts (Fig. 3K and L). These results suggest that cell clustering in slowly relaxing and highly connected matrices is not sufficient for promoting differentiation. NPCs require matrices with fast relaxation times and low connectivity to form single cell networks that can efficiently differentiate.

**Fig. 4:**
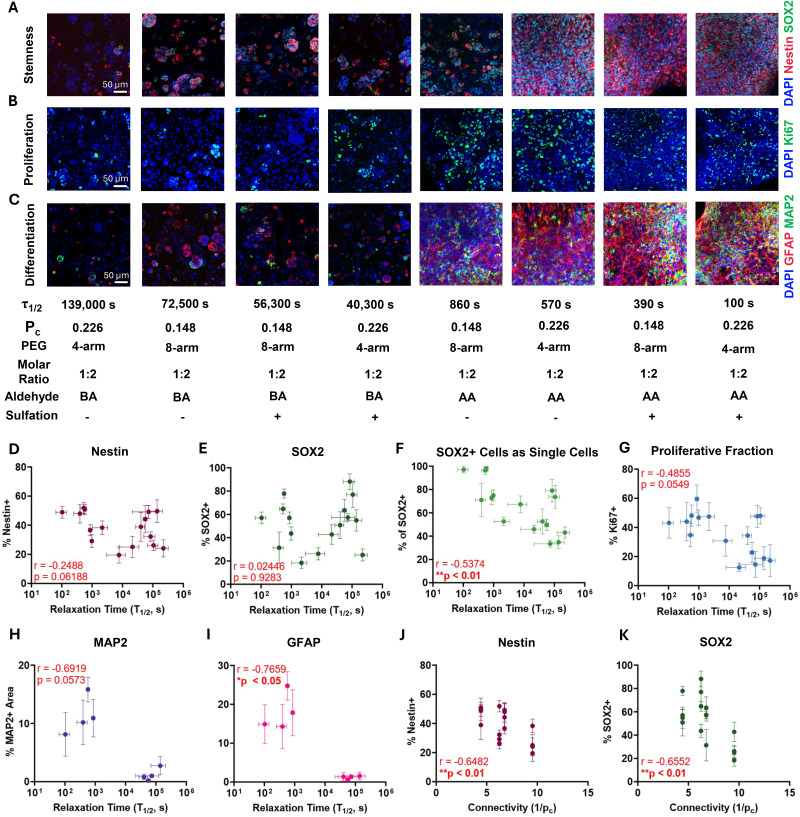
Decreasing stress relaxation timescale and connectivity promote single cell spreading and differentiation into neurons and astrocytes. Representative maximum projection fluorescence images of NPCs encapsulated in hydrogels with varying stress relaxation half-times (*τ*_1/2_) at 7 days of culture stained for (A) neural stem cell markers Nestin (red) and SOX2 (green) and DAPI (blue), (B) the proliferation marker Ki67 (green) and DAPI (blue), and (C) the neuronal marker MAP2 (green), the astrocytic marker GFAP (red), and DAPI (blue). The percentages of (D) Nestin+ and (E) SOX2+ cells are not correlated with hydrogel stress relaxation half-time. (F) The percentage of SOX2+ cells that are single cells *vs.* in clusters is correlated with faster stress relaxation half-times. (G) The percentage of Ki67+ cells displays a monotonic relationship, reaching a maximum at fast stress relaxation half-times. The percentage of (H) MAP2+ and (I) GFAP+ area increases with faster stress relaxation half-times. The percentages of (J) Nestin+ and (K) SOX2+ cells are correlated with decreased connectivity. Statistical analyses performed as Pearson correlation tests (D)–(K). *p < 0.05, **p < 0.01. Data plotted as mean ± standard error.

Among the various hydrogel properties assessed, fast stress relaxation timescales stand out as a critical factor influencing NPC phenotype. Likely due to the ability of the NPCs to maintain cell–cell contacts in hydrogels that promote formation of cell clusters, immunostaining results demonstrate that the fractions of cells positive for the stemness markers Nestin and SOX2 do not exhibit a significant correlation with stress relaxation timescales in the range tested (*p* > 0.05, Pearson rank correlation) (Fig. 4A, D and E). However, the percentage of SOX2 positive cells within the hydrogels as distributed single cells increased significantly with decreasing stress relaxation times (*p* < 0.01, Pearson rank correlation) (Fig. 4A and F). Proliferative fraction displays a monotonic trend where at fast stress relaxation times (∼10^2^–10^3^ s) cells exhibit highest proliferative fractions (Fig. 4B and G). Stemness maintenance includes both self-renewal and differentiation capacity. Both GFAP and MAP2 display a strong correlation with stress relaxation, exhibiting higher expression levels in hydrogels that relax faster (*p* < 0.05, GFAP, Pearson rank correlation).

A limitation of 3D hydrogel systems is that stress relaxation and connectivity cannot be fully decoupled. To quantify connectivity, the critical percolation threshold was calculated for each network architecture using the Flory–Stockmayer equation (see Note S1). As increasing connectivity corresponds to decreasing percolation threshold, the inverse of the percolation threshold was used to represent connectivity. Stemness marker expression displays a significant correlation (*p* < 0.05, Pearson rank correlation) with network connectivity (Fig. 4J and K). NPCs embedded in lower connectivity gels express higher levels of Nestin and SOX2 (Fig. 4J and K). The percentage of SOX2 positive cells as single cells, proliferative fraction, MAP2 positive area and GFAP positive area do not show a significant correlation with matrix connectivity (Fig. S12).

While noteworthy trends were observed as a function of stress relaxation timescale and connectivity, plotting the aforementioned markers against storage modulus revealed no significant correlation with hydrogel stiffness (*p* > 0.05, Pearson rank correlation) (Fig. S11A–F), consistent with prior work using proteolytically remodelable hydrogels.^16^ To further consider the influence of all three variables, multiple correlation analyses were used to predict cell phenotype based on hydrogel stiffness, stress relaxation timescale, and network connectivity. Nestin and SOX2 revealed a significant correlation with connectivity (*β*_3_, *p* < 0.05) whereas Ki67 (*β*_2_, *p* < 0.05) and SOX2+ single cells revealed a significant correlation with stress relaxation (*β*_2_, *p* < 0.01). No correlation was observed with stiffness (*β*_1_, *p* > 0.05) (Tables S6–S9). Together, these results demonstrate that a combination of fast stress relaxation timescale, low connectivity, and integrin-binding adhesive ligands is sufficient to maintain full NPC stemness and differentiation capacity.

**Fig. 5:**
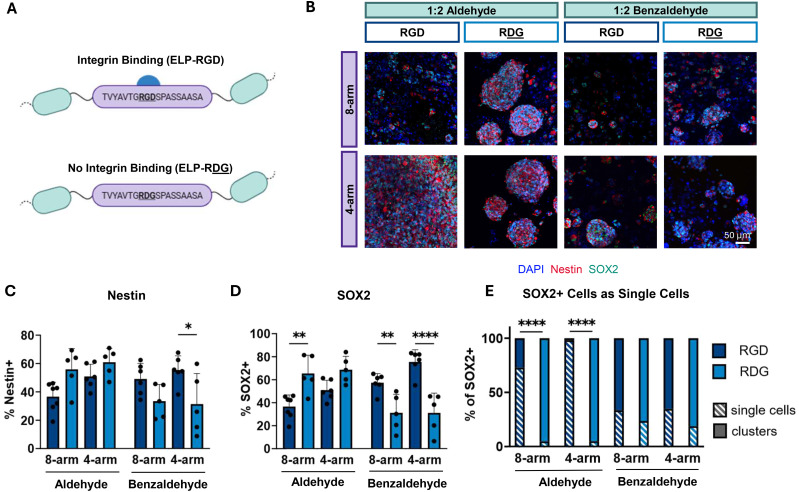
Distribution as single-cell networks, but not stemness marker expression, requires engineered integrin-binding ligands. (A) Schematic of integrin-binding RGD and scrambled non-integrin-binding RDG containing ELP sequences. (B) Representative maximum projection fluorescence images of NPCs encapsulated in aliphatic aldehyde or benzaldehyde crosslinked hydrogels with 1 : 2 hydrazine : aldehyde stoichiometry after 7 days of culture stained for neural stem cell markers Nestin (red) and SOX2 (green) and DAPI (blue). Quantification of the percentage of (C) Nestin+ and (D) SOX2+ cells in integrin-binding or non-integrin-binding hydrogels after 7 days in culture (*n* = 5–7). (E) Quantification of the percentage of SOX2+ cells that are in clusters *vs.* single cells in integrin binding or non-integrin binding hydrogels after 7 days in culture (*n* = 5–7). Statistical analyses performed as two-way ANOVA with Bonferroni multiple comparisons test (C)–(E). *p < 0.05, **p < 0.01, ****p < 0.0001. Data plotted as mean ± standard deviation. Confidence intervals and *p*-values are reported in Tables S10 (C), S11 (D) and S12 (E).

An outstanding question is whether integrin-binding ligands are necessary to promote stemness maintenance in physically remodelable, stress-relaxing hydrogels. Prior work using proteolytically remodelable hydrogels demonstrated that RGD ligands were not necessary to maintain expression of Nestin and SOX2 mRNA. We hypothesize that in physically remodelable systems, RGD engagement will be necessary to enable the cells to physically reorganize the polymer networks to enable spreading and distribution as single-cell networks. To test this hypothesis, NPCs were encapsulated in hydrogel networks comprised of a non-integrin binding ELP with a scrambled RDG bioactive site (Fig. 5A). To cover a broad range of stress relaxation timescales, hydrogels crosslinked with either aliphatic aldehyde or benzaldehyde PEGs with a 1 : 2 stoichiometric imbalance of reactive groups were used. Immunostaining on day 7 revealed that cells embedded in non-adhesive RDG hydrogels can maintain relatively high levels of stemness marker expression (Fig. 5B). Compared to adhesive RGD hydrogels, Nestin and SOX2 levels were lower in non-adhesive benzaldehyde-crosslinked gels (Fig. 5C and D). Interestingly, the percentage of Nestin and SOX2 positive cells actually increased in non-adhesive aldehyde gels (Fig. 5C and D). Although NPCs retained stemness marker expression in non-adhesive RDG hydrogels, the percentage of SOX2 positive cells as single cells significantly decreased in almost all conditions compared to their cell-adhesive counterpart (Fig. 5E). Together, these findings indicate that incorporation of a cell-adhesive ligand is necessary for distributed single-cell networks of NPCs to form in fast relaxing materials, but the adhesive ligands are otherwise dispensable for retaining stemness marker expression.

**Fig. 6:**
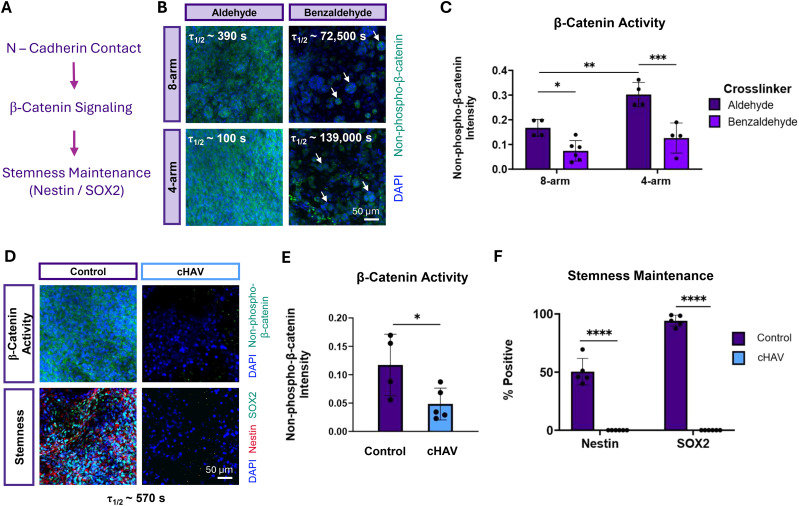
Cadherin contacts mediate β-catenin signaling and stemness maintenance. (A) Schematic detailing the mechanism of cadherin signaling mediating downstream β-catenin signaling and stemness maintenance (B) Representative maximum projection fluorescence images of NPCs encapsulated in aliphatic aldehyde or benzaldehyde crosslinked hydrogels with 1 : 2 hydrazine : aldehyde stoichiometry after 7 days of culture stained for active non-phospho-β-catenin (green) and DAPI (blue). White arrows denote high non-phospho-β-catenin staining in cell clusters. Quantification of the intensity of (C) non-phospho-β-catenin cells in hydrogels after 7 days in culture (*n* = 4–6). (D) Representative maximum projection fluorescence images of NPCs in 1 : 2 non-sulfated ELP-RGD-hydrazine + 4-arm PEG-aldehyde gels treated with a cadherin-blocking cyclic-HAV peptide (cHAV) or vehicle control (DMSO). Quantification of the intensity of (E) β-catenin activity and percentage of (F) Nestin+ and SOX2+ cells after 7 days in culture (*n* = 4–6). Statistical analyses performed as lognormal unpaired *t*-test (E) and two-way ANOVA with Bonferroni multiple comparisons test (C) and (F). *p < 0.05, **p < 0.01, ***p < 0.001 ****p < 0.0001. Data plotted as mean ± standard deviation. Confidence intervals and *p*-values are reported in Tables S13 (E) and S14 (F).

This raises the question of how NPCs maintain stemness in matrices without adhesive ligands. Prior work has demonstrated that murine NPCs require matrix remodeling to form N-cadherin contacts that influence downstream β-catenin signaling and stemness maintenance^16^ (Fig. 6A). We postulated that the cell clustering observed in slowly relaxing cell-adhesive matrices and rapidly relaxing non-adhesive ELP-RDG matrices serves as a way for NPCs to maintain cell–cell contacts and stemness. To validate this mechanism, NPCs were embedded in four different hydrogel conditions with varying stress relaxation timescales and connectivity. Non-phospho-β-catenin (active β-catenin) staining revealed robust and distributed staining in rapidly relaxing matrices. However, in slowly relaxing matrices, similar staining was localized only to cell clusters (Fig. 6B). Non-phospho-β-catenin intensity was significantly higher in aliphatic aldehyde gels compared to benzaldehyde conditions, and in 4-arm compared to 8-arm aliphatic aldehyde conditions (Fig. 6C). These data suggest that cell clusters enable cell–cell contacts and downstream β-catenin signaling. To demonstrate that cell–cell contacts are required for NPC stemness maintenance in rapidly relaxing, low connectivity hydrogels, NPCs were treated with a cadherin-blocking cyclic-HAV peptide (cHAV), as previously described.^11,16^ Immunostaining revealed the loss of both β-catenin activity and stemness marker expression in NPCs treated with the cHAV peptide (Fig. 6D). Compared to vehicle control, cadherin-blocked samples displayed significantly lower levels of β-catenin activity and Nestin and SOX2 expression (Fig. 6E and F). These results suggest that cadherin contacts are necessary for maintaining stemness in human NPCs in 3D hydrogel networks.

## Discussion

Expansion of functional neural progenitor cells remains a barrier to translation of cell therapies that may be addressed by engineering appropriate materials systems to support NPC culture. Prior results using murine brain-derived NPCs indicated that remodeling of hydrogel matrices was essential to maintain stemness by enabling cell–cell contact and β-catenin signaling.^16–18^ The present results extend this observation to human brain-derived NPCs. In the prior murine studies, either proteolytic remodeling of protein or peptide-crosslinked hydrogels or physical remodeling mediated by rapid stress relaxation of ionically crosslinked alginate gels was sufficient to maintain stemness.^16^ More recent work utilizing hybrid protein–hyaluronic acid hydrogels demonstrated that intermediate timescales of stress relaxation could similarly promote stemness maintenance, but the inclusion of N-cadherin mimetic binding sites was required to achieve the same phenotypes seen in highly remodelable systems.^11^ The present study reconciles these prior observations by identifying stress relaxation timescale and matrix connectivity as two critical design elements. By carefully tuning crosslinker chemistry, network architecture, and polymer phase separation behavior, engineered protein hydrogels with stress relaxation timescales on the order of native brain tissue (∼10^2^ s) were obtained. At such rapid stress relaxation timescales, inclusion of only an integrin-binding cell adhesive motif was required for stemness maintenance as single cells, consistent with the prior studies in alginate gels with similar relaxation timescales.^16^

At slower stress relaxation timescales, NPCs still maintained a high degree of stemness marker expression in hydrogels with low degrees of connectivity, achieved by using crosslinkers with lower functionality or a stoichiometric imbalance of reactive groups. As proteolytic remodeling of ELP-based hydrogels previously supported murine NPC stemness maintenance, we propose that a similar effect is observed in the present study with human NPCs. In purely elastic ELP–PEG hydrogel systems with irreversible covalent crosslinks, tuning the functionality of the PEG crosslinkers predictably regulated network degradability and, in turn, cell spreading and cellular organization.^22,35^ With proteolytic degradation confined primarily to the bioactive domains of the ELPs, the degradability of the network is correlated with the degraded network connectivity, as expressed by the critical percolation threshold for the proteolyzed materials derived from the Flory–Stockmeyer equation.^22^ As connectivity decreases, the degradability and susceptibility to proteolytic remodeling increases. To quantify degradability in the ELP–PEG systems, the critical percolation thresholds for the degraded networks were divided by the calculated extent of reaction for each condition (Table S15). As the extent of reaction approaches the critical threshold, the network becomes more susceptible to degradation. Degradability is maximized in systems with bifunctional crosslinkers, and accordingly, encapsulating human NPCs in ELPs crosslinked by irreversible isonitrile ligations using bifunctional PEGs also resulted in stemness maintenance, even in non-relaxing hydrogels (Fig. S13A and B).^35^ Stemness maintenance is significantly correlated with degradability across the various ELP–PEG formulations assessed (Fig. S13C–F).

**Fig. 7:**
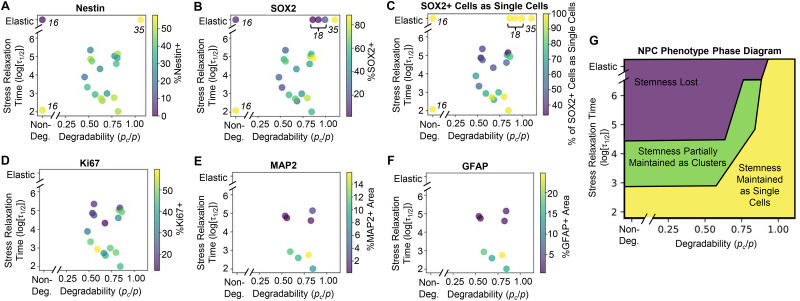
Stress relaxation timescale and network degradability regulate NPC phenotype. Plots of stress relaxation *versus* calculated network degradability overlaid with percentage of (A) Nestin+ cells, (B) SOX2+ cells, and (C) SOX2+ cells as single cells, and (D) Ki67+ cells, and percentage of area positive for (E) MAP2 and (F) GFAP, aggregating data from this study and prior studies using murine and human brain-derived NPCs.^16,18,35^ The numbers adjacent to the data points correspond to the reference from which the data were obtained. (G) Proposed phase diagram relating hydrogel stress relaxation timescale and proteolytic degradability to NPC phenotype.

To propose design guidelines for hydrogel systems to maintain NPC stemness, we identified related datasets that included non-degradable viscoelastic hydrogel systems and proteolytically degradable ELPs with irreversible crosslinks that confer elastic mechanical properties.^16,18^ Only studies using the same ELPs as the present study were included. Studies that included enzymatically degradable crosslinkers (*e.g.*, hyaluronic acid) were excluded to ensure degradability could be compared across systems. Further, only studies using brain-derived NPCs that are capable of differentiation into neurons and astrocytes were included.^36,37^ A resulting caveat is that the cells compared are derived either from mice (ref. 16 and 18) or humans (ref. 35 and the present study). By comparing metrics of stemness across this expanded dataset, a phase diagram can be plotted summarizing the roles of degradability and stress relaxation in regulating neural progenitor cell phenotype (Fig. 7). In aggregate, permitting high degrees of remodeling, whether through proteolysis or rapid stress relaxation, can robustly maintain NPC function in three-dimensional cultures.

## Conclusion

This work presents a hydrogel system in which network connectivity, stress relaxation timescale, polymer phase separation, and adhesive ligand presentation can be simultaneously tuned. Embedding NPCs into viscoelastic matrices with low connectivity, fast stress relaxation timescales, and the presence of the RGD integrin binding motif mediated NPC stemness maintenance, proliferation, and differentiation. Mechanistic experiments revealed cell clustering promotes cell–cell contacts, β-catenin signaling and stemness maintenance irrespective of adhesive ligand inclusion. Engineering rapidly relaxing matrices that successfully promote NPC function is thus an attractive strategy to culture NPCs *ex vivo*, with potential applications in expansion of cells for regenerative medicine approaches or *in vitro* disease modeling and drug screening.

## Materials and methods

### Materials and characterization

All water sensitive reactions were completed with commercially available anhydrous solvents. PEG-aldehyde (MW: 10 kDa, 4arm) was purchased from creative PEGWorks and multi-arm PEG-amine precursors were purchased from JenKem Technology. Benzaldehyde-NHS ester was synthesized as described previously.^38^ All other reagents were purchased from Thermo Fisher Scientific or Millipore Sigma and used as received, unless otherwise noted. Rheological characterization was performed on a Rheometrics ARES-RFS3 strain-controlled rheometer. Confocal microscopy images were taken with a Zeiss LSM 900 laser scanning confocal microscope equipped with a broadband (385–730 nm) widefield epifluorescence light source and a Photometrics Prime BSI camera. Silicone molds used in 3D cell culture were plasma bonded onto circular glass coverslips using an Anatech SCE-106 Barrel Asher located in the Singh Center for Nanotechnology and subsequently sterilized with 70% ethanol. ^1^H NMR spectra were collected on a Bruker NEO 600 spectrometer at 600 MHz. Chemical shifts were referenced to the residual solvent peak (CDCl_3_, *δ*^1^H = 7.26 ppm, *δ*^13^C = 77.16 ppm, DMSO-*d*_6_, *δ*^1^H = 2.50 ppm, *δ*^13^C = 39.52 ppm, D_2_O, *δ*^1^H = 4.79 ppm) and denoted in parts per million (ppm).

### Expression and characterization of ELPs

ELPs used were developed by Straley and Heilshorn.^25^ As previously reported, both RGD (cell adhesive) and RDG (non-cell adhesive) were cloned into a pET15b plasmid with a corrected T7 promoter site (pET15b-T7pCONS sfGFP) *via* Addgene (#154467) courtesy of Prof. Daniel Daley (University of Stockholm).^35^ Briefly, plasmids containing ELP-RGD and ELP-RDG were transformed into BL21(DE3)pLysS *Escherichia coli*. The ELP genes were expressed under the control of the T7 promoter. Bacteria were cultured in Terrific Broth at 37 °C until an OD600 of 0.6, at which point the temperature was reduced to 32 °C. At an OD600 of 0.8, 1 mM isopropyl β-d-1 thiogalactopyranoside (IPTG) was added to induce expression. The protein was afforded 7 hours to express before collecting the bacteria by centrifugation. Bacterial pellets were resuspended in TEN buffer (10 mM Tris, 1 mM EDTA, and 100 mM NaCl, pH 8.0) and lysed using a series of repetitive freeze–thaw cycles. DNAse1 and phenylmethylsulphonyl fluoride (PMSF) were added once to the raw lysate to remove genomic DNA and inhibit proteases. The thawed lysate was adjusted to a pH of 9.0 using NaOH and purified by inverse temperature cycling at 37 °C and 4 °C.^39^ ELP solutions were desalted by dialyzing against MilliQ water for 48 hours (4 × 4.5 L, 4 °C). Dialyzed solutions were frozen in liquid nitrogen and lyophilized for 72 hours to afford a white solid. Expression of ELP-RGD and ELP-RDG yielded 1.72 g and 1.37 g, respectively, from 12 L of bacterial culture. Protein purity was confirmed post-expression by SDS-PAGE (Fig. S14).

### Synthesis and characterization of cysteic acid-functionalized ELP

ELPs were functionalized with a cysteic acid moieties on the primary amines of the recombinant proteins, based on a previously published procedure.^34^ The ELP (MW: 38 kDa, 0.2 g) was fully dissolved in anhydrous dimethyl sulfoxide (DMSO) at room temperature, and an equivalent amount of anhydrous dimethylformamide (DMF) was added. In a separate round bottom flask, Boc-l-cysteic acid (Boc-CysA; 4.2 eq. per amine, BLDpharm) and HATU (4 eq. per amine, Advanced Chemtech) were fully dissolved in DMF at room temperature. Afterwards, *N*,*N*-diisopropylethylamine (DIEA, 10 eq. per amine) was added to the Boc-CysA:HATU:DMF reaction vessel and left to stir for 10 minutes. The activated mixture was added dropwise to the ELP solution and allowed to react overnight at room temperature.

The product was precipitated by dropwise addition into ice-cold diethyl ether, collected by centrifugation (25 min, 4 °C, 18 000 RCF), and dried under argon gas. A small quantity (∼10 mg) was collected to quantify reaction efficiency *via* NMR analysis. To remove the Boc protecting group, the dried ELP sample was dissolved in 6 mL of a 1 : 1 (v : v) mixture of dicloromethane (DCM) : trifluoroacetic acid (TFA) plus triisopropylsilane (TIPS, 100 µL) and MilliQ-grade water (100 µL) as scavengers. The reaction mixture was stirred for 4 hours at room temperature. The deprotected ELP was precipitated by dropwise addition into ice-cold diethyl ether, collected by centrifugation, and dried under argon gas. A small quantity (∼10 mg) was collected to confirm deprotection *via* NMR analysis. Dried ELP was dissolved in MilliQ-grade water and dialyzed against MilliQ-grade water for 48 hours (4 × 4.5 L, 4 °C). Dialyzed solutions were frozen in liquid nitrogen and lyophilized for 72 hours to afford a white solid. ^1^H NMR characterization is provided in Fig. S5.

### Synthesis and characterization of hydrazine-functionalized ELP

ELPs were functionalized with hydrazine moieties on the primary amines of the proteins, following established methods.^21,40,41^ Briefly, ELP (MW: 38 kDa, 0.4 g) was fully dissolved in anhydrous DMSO at room temperature, and an equivalent amount of anhydrous DMF was added. In a separate round bottom flask, tri-Boc-hydrazinoacetic acid (4.2 eq. per amine) and HATU (4 eq. per amine, Advanced Chemtech) were fully dissolved in DMF at room temperature. Afterwards, DIEA (10 eq. per amine) was added to the HATU-containing reaction vessel, and the mixture was stirred for 10 minutes. The activated mixture was added dropwise to the ELP mixture and allowed to stir overnight at room temperature.

The product was precipitated by dropwise addition into ice-cold diethyl ether, collected by centrifugation (25 min, 4 °C, 18 000 RCF), and dried under argon gas. A small quantity (∼10 mg) was collected to quantify reaction efficiency *via* NMR analysis. To remove the Boc protecting group, the dried ELPs were dissolved in 6 mL of a 1 : 1 (v : v) mixture of DCM : TFA plus triisopropylsilane (TIPS, 100 µL) and MilliQ-grade water (100 µL) as scavengers. The reaction mixture was stirred for 4 hours at room temperature. The deprotected ELP was precipitated by dropwise addition into ice-cold diethyl either, collected by centrifugation, and dried under argon gas. A small quantity (∼10 mg) was collected to confirm deprotection *via* NMR analysis. Dried ELP was dissolved in MilliQ-grade water and dialyzed against MilliQ-grade water for 48 hours (4 × 4.5 L, 4 °C). Dialyzed solutions were frozen in liquid nitrogen and lyophilized for 72 hours to afford a white solid. ^1^H NMR characterization is provided in Fig. S1.

### ELP lower critical solution temperature characterization

Lower critical solution temperature (LCST) transition temperature (*T*_*t*_) measurements were performed using a circular dichroism (CD) spectrometer (J-1500, JASCO). Optical density measurements of ELP solutions were collected (*λ* = 300 nm) following previously established protocols.^23,41–43^ ELPs were dissolved at a 1% concentration (w/v; 10 mg mL^−1^) in PBS overnight at 4 °C. ELP solutions (200 µL) were maintained on ice and loaded into a 1 mm path length quartz cuvette and compared against PBS blanks. Absorbance readings were collected (*λ* = 300 nm) at increments of 1 °C spanning from 4 °C to 65 °C. The temperature was allowed to stabilize for 10 s prior to each measurement. Reported curves are baseline corrected and normalized. Reported transition temperatures are the temperature at which the normalized absorbance was equal to 0.5.

### Synthesis and characterization of benzaldehyde-functionalized PEG

Multi-arm PEG-amine HCl salts (MW: 10 kDa, 4arm; MW: 20 kDa, 8arm) were lyophilized overnight to draw out any remaining moisture. PEG-amines and benzaldehyde-NHS ester (2 eq. per amine) were added to a round bottom flask and dissolved in anhydrous DMF. Afterwards, DIEA (4 eq. per amine) was added to the reaction vial and left to stir overnight at room temperature.

The product was precipitated by dropwise into ice-cold diethyl ether, collected by centrifugation (25 min, 4 °C, 18 000 RCF), and dried under argon gas. A small quantity (∼10 mg) was collected to quantify reaction efficiency *via* NMR analysis. The dried PEGs were dissolved in MilliQ-grade water and dialyzed against MilliQ-grade water for 48 hours (4 × 4.5 L, 4 °C). Dialyzed solutions were frozen in liquid nitrogen and lyophilized for 72 hours to afford a yellowish-white solid. ^1^H NMR characterization is provided in Fig. S15.

### Synthesis and characterization of aliphatic aldehyde-functionalized PEG

Multi-arm aliphatic aldehyde PEG crosslinkers were synthesized by Swern oxidation based on a published procedure.^28^ A solution of oxalyl chloride (1.5 mL, 17.6 mmol, 11 eq. per hydroxyl) in anhydrous DCM (20 mL) was added to a round bottom flask equipped with an addition funnel under an argon atmosphere and cooled in a dry ice/acetone bath. A solution of DMSO (1.3 mL, 18.5 mmol, 11.5 eq.) in anhydrous DCM (6.5 mL) was added dropwise *via* the addition funnel over the course of 5 minutes and allowed to react for an additional 10 minutes. 8-arm 20 kDa PEG–OH (4 g, 0.2 mmol) was dissolved in anhydrous DCM (5 mL) and added dropwise *via* the addition funnel over the course of 10 minutes. The reaction was allowed to proceed for an additional 2 hours. Triethylamine (5.6 mL, 40 mmol, 25 eq.) was added dropwise *via* the addition funnel over the course of 10 minutes and reacted for an additional 20 minutes. The reaction mixture was allowed to warm to room temperature, and the product was precipitated in ice-cold diethyl ether and collected by centrifugation. The resulting solid was dried under argon, redissolved in MilliQ-grade water, and dialyzed against MilliQ-grade water for 48 hours (4 × 4.5 L, 4 °C). Dialyzed solutions were frozen in liquid nitrogen and lyophilized for 72 hours to afford a white solid. ^1^H NMR characterization is provided in Fig. S16.

### Hydrogel formation

ELP and PEG precursor solutions were dissolved in PBS at 4 °C overnight and maintained on ice until used. The precursor concentrations were chosen to maintain a 1 : 1 or a 1 : 2 ratio of hydrazine to aldehyde reactive groups. ELP–PEG hydrogels were prepared by mixing two precursor solutions in a 1 : 1 (v : v) ratio. Hydrogel compositions are detailed in Table S16.

### Rheological characterization

Oscillatory rheology was performed on hydrogel precursor solutions prepared as described above using a 25 mm 0.02-radian cone geometry. Gel precursor solutions were gelled *in situ* on a Peltier stage at 4 °C to prevent protein aggregation before gelation. Time sweeps were performed for 1 hour at 22 °C at 5% strain and 1 Hz oscillatory frequency. Temperature ramps from 22 °C to 37 °C were performed at 5% strain. Frequency sweeps were performed at a constant 3% strain and oscillatory frequency ranging from 0.1 Hz to 10 Hz at 37 °C (Fig. S17). Strain sweeps were performed at constant oscillatory frequency of 1 Hz and initial and final strains of 0.1% and 10%, respectively, at 37 °C (Fig. S18). Stress relaxation tests were completed under a constant strain of 10% at 37 °C. Stress relaxation half-times for PEG–aldehyde crosslinked hydrogels were taken from measured stress relaxation *τ*_1/2_ values (Fig. S19 and S20). Double Maxwell models (two-phase decay) were used to predict *τ*_1/2_ values for PEG–benzaldehyde crosslinked hydrogels that do not relax in a tractable amount of time (Fig. S21 and S22). Storage moduli were reported as the average value around 1 Hz oscillatory frequency from the frequency sweeps. Confidence intervals and *p*-values are reported in Table S17.

### Cell culture and encapsulation

Human neural progenitor cells (ReNcell CX, Millipore Sigma) were cultured and expanded in poly-l-ornithine- and laminin-coated tissue culture flasks in maintenance medium (ReNcell medium supplemented with 20 ng mL^−1^ FGF-2 and 20 ng mL^−1^ EGF). In preparation for hydrogel encapsulation, cells were detached with Accutase, collected by centrifugation, and counted using a hemocytometer. The cells were resuspended in the ELP pre-gel solutions to achieve a final cell density of 25 × 10^6^ mL^−1^. A 1 : 1 (v : v) ratio of PEG–aldehyde solution was added to crosslink the hydrogels, wherein the PEG concentration and composition were varied to achieve the desired network properties. Gels were cast within custom silicone molds and subsequently covered with maintenance medium. To assess stemness marker expression and proliferation capacity, NPCs were cultured in maintenance medium for seven days. To assess differentiation capacity, NPCs were first cultured for 7 days in maintenance medium before differentiation was induced by withdrawing growth factor supplementation and culturing for an additional 7 days.

### Immunofluorescence analysis

For immunocytochemistry, NPC-containing hydrogels were fixed with 4% paraformaldehyde in PBS for 30 min at 37 °C. Samples were permeabilized with 0.25% Triton X-100 in PBS (PBST) for 1 hour at room temperature and blocked with 5% bovine serum albumin (BSA) and 5% goat serum (GS) in PBST for 3 hours at room temperature. The samples were incubated with primary antibodies (Table S18) diluted in PBST containing 2.5% BSA and 2.5% GS overnight at 4 °C on an orbital shaker. Samples were washed a total of three times with PBST at room temperature and subsequently incubated with the desired secondary antibodies (Table S19) and DAPI diluted in PBST containing 2.5% BSA and 2.5% GS overnight at 4 °C on an orbital shaker. Samples were sufficiently washed with PBST at room temperature and mounted using Fluoromount-G^TM^ Slide Mounting Media (SouthernBiotech). Samples were imaged by confocal microscopy. Cells positive for Nestin, SOX2, Ki67, calcein-AM and ethidium-homodimer were manually counted using FIJI ImageJ software and reported as percentage of total cells. Cell clusters were defined as four or more cells touching or within 10 µm of each other and were manually counted. The percentage of SOX2+ cells as single cells is reported relative to total SOX2+ cells. Differentiation marker (MAP2 and GFAP) expression was reported as percent area positive for that marker calculated using ImageJ. Non-phospho-β-catenin intensity was measured using CellProfiler.

### N-Cadherin inhibition

NPCs were detached with Accutase and incubated in maintenance medium with 1 mg mL^−1^ cHAV peptide (Exherin, AdooQ Bioscience) with constant agitation for 30 min at at 37 °C. Treated NPCs were collected by centrifugation and encapsulated as described above. Cells were cultured in maintenance media supplemented with 1 mg mL^−1^ cHAV peptide for 7 days. Media was replaced every other day supplemented with 1 mg mL^−1^ cHAV peptide. NPCs were treated with DMSO served as vehicle controls.

### Cell viability

Cells were encapsulated as described above and incubated at 37 °C. After 24 hours, maintenance media was changed and supplemented with 1 µM calcein-AM and 1 µM ethidium-homodimer. The samples were incubated at 37 °C for 45 min and washed twice with maintenance media. Live samples were imaged by confocal microscopy (Fig. S3).

### Statistical analysis

A minimum of 3 independent replicates were used for each experiment. Statistical analysis was performed using GraphPad Prism 10. Comparisons between two experimental groups were analyzed using a lognormal unpaired *t*-test. Comparisons among more than two experimental groups with two varying parameters were analyzed by a two-way ANOVA with Bonferroni *post hoc* testing. To model the relationships between dependent variables of interest (Nestin, SOX2, SOX2+ single cells, Ki67) against independent variables (stiffness, stress relaxation half-time, and connectivity) multiple linear regressions were used. To determine correlation, Pearson correlation tests were performed. n.s. = not significant (*p* > 0.05), *p < 0.05, **p < 0.01, ***p < 0.001, ****p < 0.0001.

## Conflicts of interest

There are no conflicts of interest to declare.

## Supplementary Material

TB-014-D5TB02537K-s001

## Data Availability

All data supporting this study are contained in the main text, supplementary information (SI), or available as a publicly accessible database *via* Zenodo (https://doi.org/10.5281/zenodo.19005159). Supplementary information (theoretical prediction of extent of reaction and degradability, supporting figures and data tables, and detailed statistical analysis information). See DOI: https://doi.org/10.1039/d5tb02537k.
