# Supplementary material for: Stress relaxation timescale and hydrogel network connectivity regulate neural progenitor cell stemness and differentiation
Source: J Mater Chem B. 2026 Apr 1;14(15):4740–54. doi: 10.1039/d5tb02537k (PMC13058797; doi:10.1039/d5tb02537k)
Supplement: TB-014-D5TB02537K-s001 [file TB-014-D5TB02537K-s001.pdf]

Supplementary Information (SI) for Journal of Materials Chemistry B.
This journal is © The Royal Society of Chemistry 2026

# Stress Relaxation Timescale and Hydrogel Network Connectivity Regulate Neural Progenitor Cell Stemness and Differentiation

Lauren Brown,[1] Daphne Bakker,[2] Ping Zhou,[2] Christopher M. Madl[2,3]*

1. Department of Bioengineering, University of Pennsylvania, Philadelphia, PA 19104 USA
2. Department of Materials Science and Engineering, University of Pennsylvania, Philadelphia, PA 19104 USA
3. Center for Precision Engineering for Health (CPE4H), University of Pennsylvania, Philadelphia, PA 19104 USA

*Corresponding author: cmadl@seas.upenn.edu

## Supporting Information

Supporting Note……………………………………………………………………………...2

Supporting Figures……………………………………………………………………………...4

Supporting Tables…………………………………………………………………………..22

Supporting References…………………………………………………………………...38

**Supporting Note S1: Theoretical prediction of extent of reaction and network degradability**

The hydrazone-based crosslinks in the system exist in dynamic equilibrium:

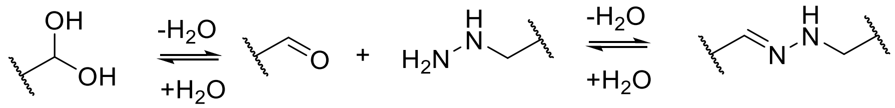


Thus, any technique to quantify extent of reaction that would require chemical reaction with unreacted crosslinking groups or that would require removal of water or nucleophiles like amines from the system will change the concentration of reactive groups participating in crosslinks.

Accordingly, to estimate the extent of reaction in the hydrogels, we relate the measured storage moduli of the hydrogels to predicted elastic moduli for full conversion.

The modulus ($G_{ideal}$) of a stoichiometrically balanced ideal network is given by:

$$G_{ideal} = C\nu_e k_B T$$

Where $k_B$ is Boltzmann's constant, $T$ is the absolute temperature, and the ideal concentration of elastic chains ($\nu_e$) follows the form derived by Miller and Macosko[1]:

$$\nu_e = \frac{\rho N_A}{M_{n,A} + \frac{f_A}{f_B} M_{n,B}} \times \frac{f_A}{2}$$

Where $\rho$ is the mass concentration of polymer in the network, $N_A$ is Avogadro's number, $M_{n,A}$ is the number average molecular weight of the A-type molecules (~38,000 Da for ELP), $f_A$ is the functionality of the A-type molecules (14 for ELP), $M_{n,B}$ is the number average molecular weight of the B-type molecules (~10,000 Da for 4-arm PEG or ~20,000 Da for 8-arm PEG), and $f_B$ is the functionality of the B-type molecules (4 or 8 for PEG).

The pre-factor $C$ can be estimated by the phantom network model[2] as:

$$C = 1 - \frac{2}{f_{avg}}$$

Where $f_{avg}$ is the average functionality of the network. For stoichiometrically balanced networks, the average functionality is:

$$f_{avg} = \frac{N_A f_A + N_B f_B}{N_A + N_B}$$

Where $N_A$ is the number of molecules with A-type reactive groups (hydrazines on ELP) and $N_B$ is the number of molecules with B-type reactive groups (aldehydes on PEG).

For stoichiometrically unbalanced mixtures, the maximal extent of reaction is dictated by the limiting reactant (hydrazines on ELP), so the equation becomes:

$$f_{avg} = \frac{2N_A f_A}{N_A + N_B}$$

Stoichiometric imbalance further impacts the calculated modulus by increasing the likelihood of dangling ends and non-elastically active chains. Empirical studies demonstrate a power law scaling dependence of $G_{ideal}$ on the stoichiometric ratio, $r$ (where $r \leq 1$),[3] with $G_{ideal}$ scaling as:

$$G_{ideal,r} \approx r^2 \times G_{ideal}$$

After the critical gelation point but before high extents of conversion, the measured modulus also follows a power law scaling relationship with the extent of reaction[4]. Thus, the measured modulus ($G$) and the calculated modulus ($G_{ideal,r}$) can be used to estimate the extent of reaction of the limiting reactant ($p$) as follows:

$$p = p_c + (1 - p_c)\left(\frac{G}{G_{ideal,r}}\right)^{1/3}$$

Where the critical extent of reaction for gelation is given by the Flory-Stockmayer equation:

$$p_c = \frac{1}{\sqrt{r(f_A - 1)(f_B - 1)}}$$

The Flory-Stockmayer equation was also used to calculate $p_{c,deg}$, the critical extent of reaction needed to maintain the gel network after proteolytic degradation. As elastin-like repeats exhibit limited protease sensitivity,[5] and prior work using murine NPCs validated that proteolytic degradation occurs primarily in the bioactive sites of the ELPs used[6] we assume that proteolysis is largely restricted to the bioactive sites in the present study. Thus, after complete degradation, the functionality ($f_A$) of an ELP molecule decreases to 3, based on the location of the lysine residues within the proteins.

The calculated extents of reaction ($p$), critical gelation points ($p_c$), and critical gelation points after degradation ($p_{c,deg}$) are presented in Supporting Table S15.

We note that this approach likely underestimates the extent of reaction for these networks. While the phantom network approach is an improvement over ideal elastomer theory, more recent work has shown that even better agreement can be obtained using approaches that account for the contribution of loops in the networks, which further suppress the measured modulus[7]. Additionally, for the off-stoichiometric networks, while percolation theory suggests a scaling of $G$ as $r^2$, empirical data reveal power law scaling with exponents as high as ~3 in some cases.[3]

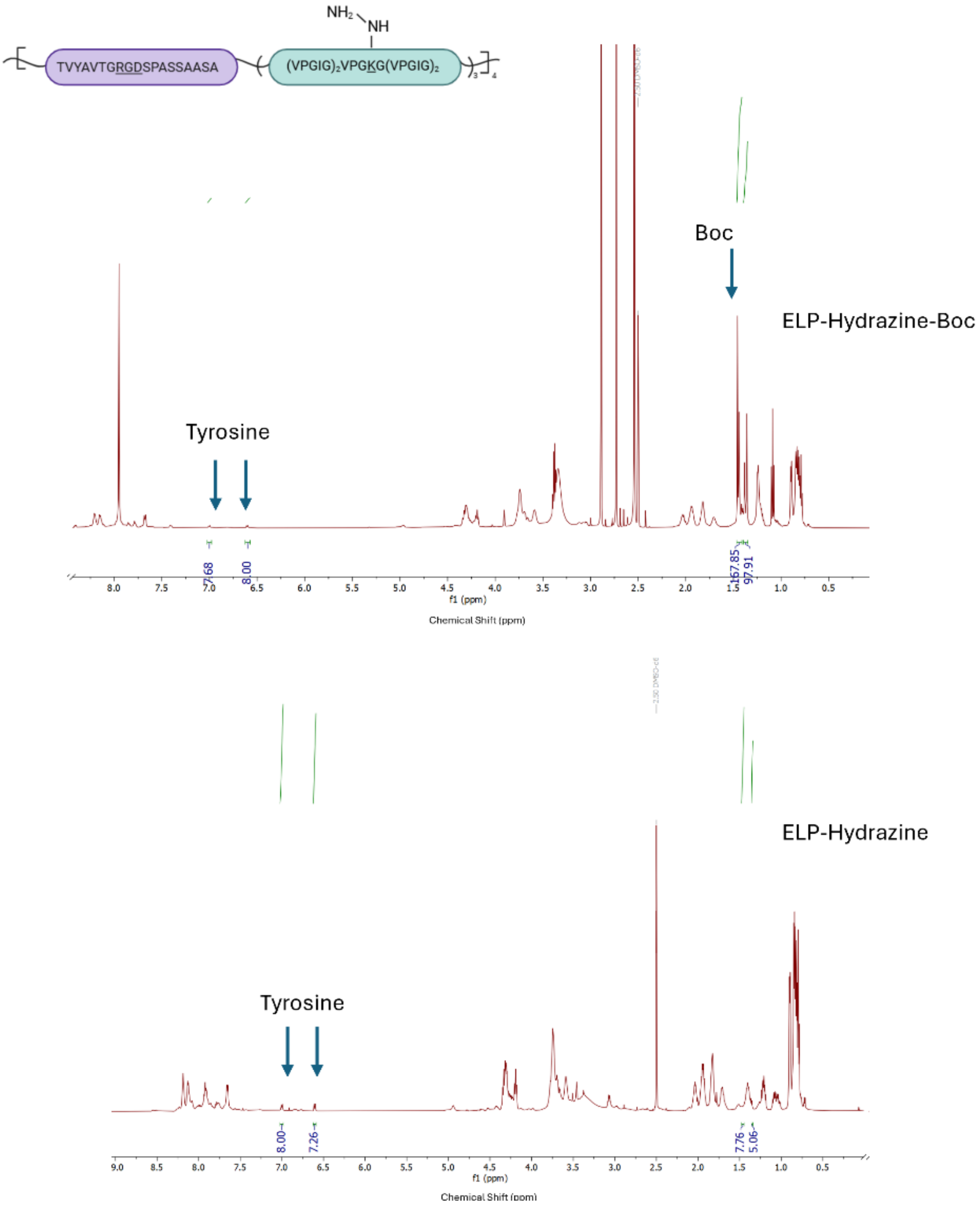


**Supplemental Figure S1.** $^{1}$H NMR of ELP-RGD-Hydrazine in DMSO-$d_6$.

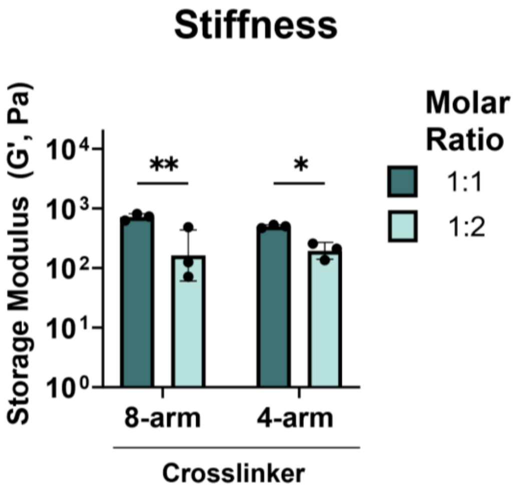


**Supplemental Figure S2.** Storage moduli of ELP-hydrazine crosslinked with aliphatic aldehyde PEGs (n = 3). Statistical analysis performed as two-way ANOVA with Bonferroni post-hoc tests. *p < 0.05, ** p < 0.01. Data plotted as mean ± standard deviation.

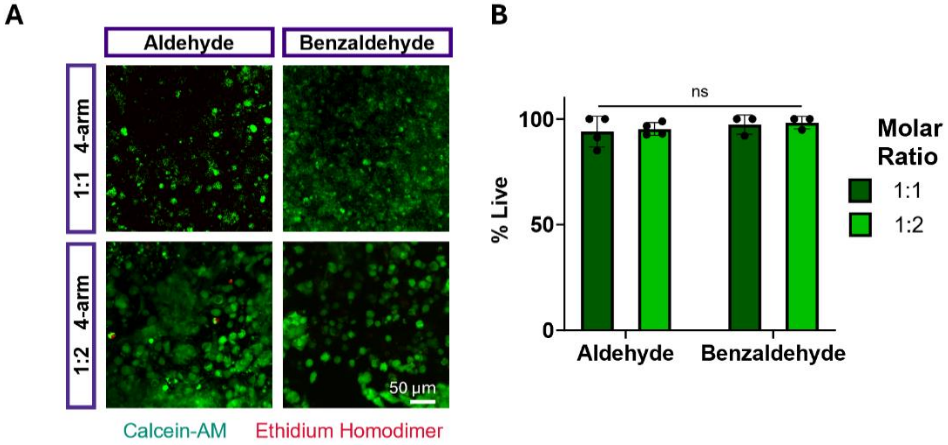


**Supplemental Figure S3. (A)** Representative maximum projection fluorescence images of NPCs stained for calcein-AM (green) and ethidium homodimer (red) after 24 hours in culture. Quantification of the percentage of **(B)** calcein-AM+ cells after 24 hours in culture.

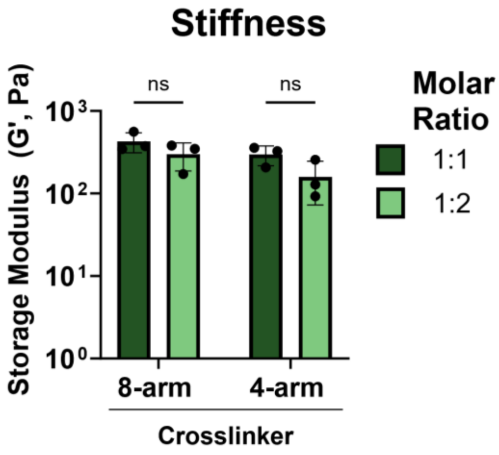


**Supplemental Figure S4.** Storage moduli of ELP-hydrazine crosslinked with benzaldehyde PEGs (n = 3). Statistical analysis performed as two-way ANOVA with Bonferroni post-hoc tests. Data plotted as mean ± standard deviation.

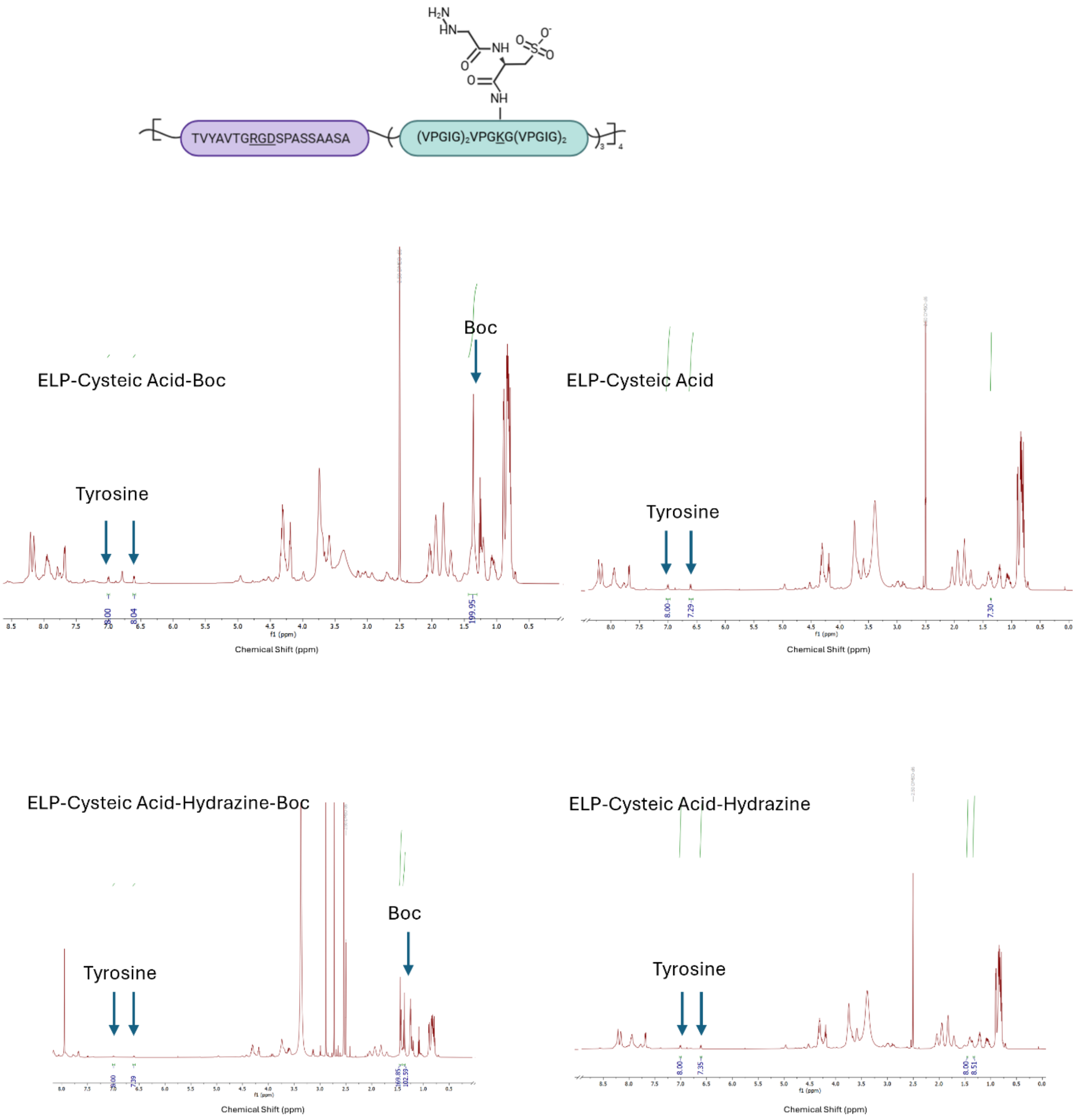


**Supplemental Figure S5.** $^{1}$H NMR of ELP-RGD-Cysteic Acid-Hydrazine in DMSO-$d_6$.

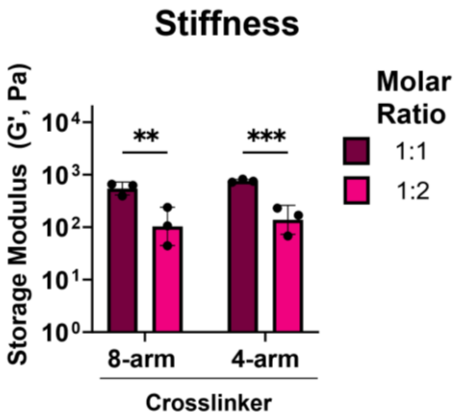


**Supplemental Figure S6.** Storage moduli of aliphatic aldehyde crosslinked sulfated ELP-hydrazine hydrogels ($n = 3$). Statistical analysis performed as two-way ANOVA with Bonferroni post-hoc tests. ** $p < 0.01$, *** $p < 0.001$. Data plotted as mean ± standard deviation.

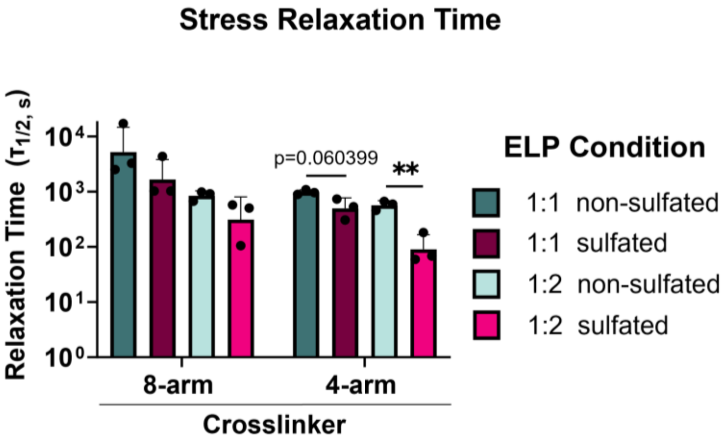


**Supplemental Figure S7.** Stress relaxation half-times ($\tau_{1/2}$) of sulfated versus non-sulfated aliphatic aldehyde crosslinked hydrogels ($n = 3$). ** $p < 0.01$. Statistical analyses performed as lognormal unpaired t-tests. Data plotted as mean ± standard deviation.

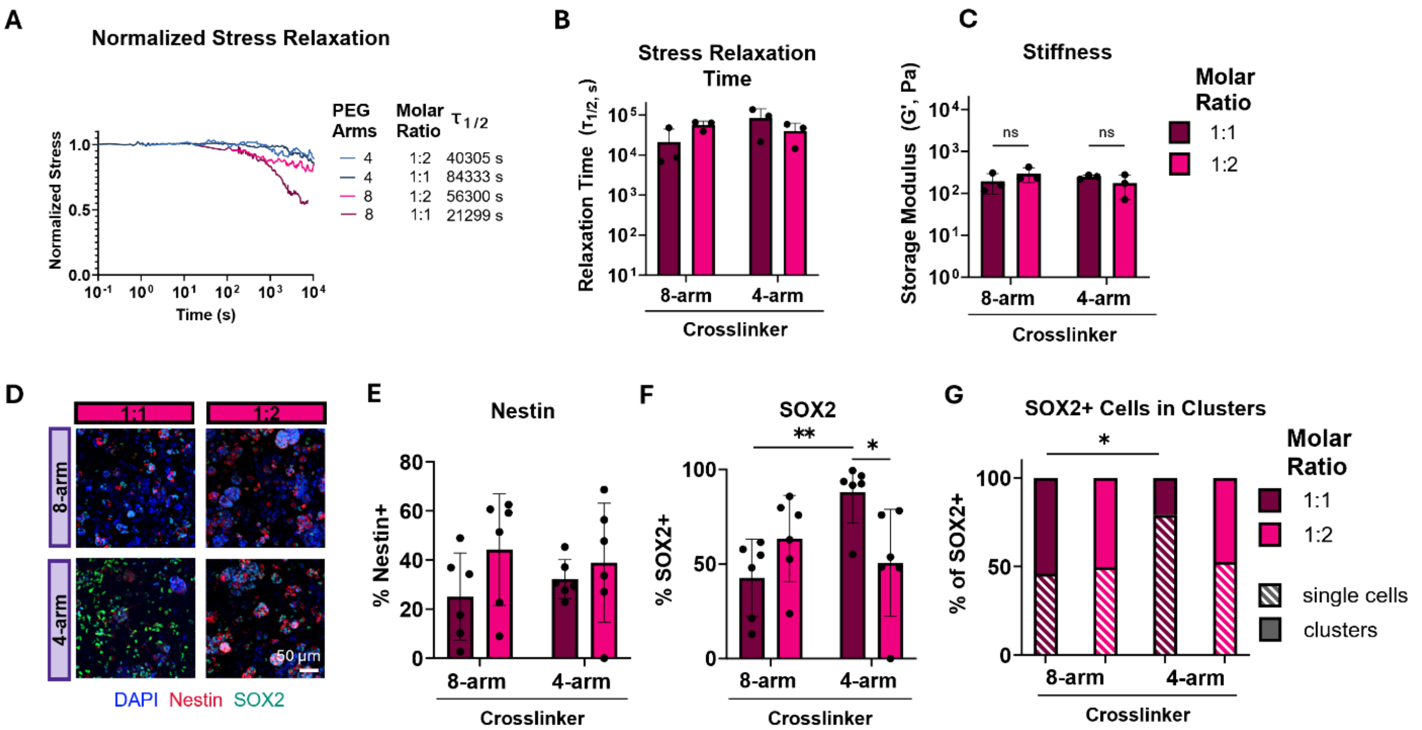


**Supplemental Figure S8. (A)** Normalized representative stress relaxation curves for benzaldehyde crosslinked sulfated hydrogels. a.u., arbitrary units. **(B)** Stress relaxation half-times ($\tau_{1/2}$) and **(C)** storage moduli for benzaldehyde crosslinked sulfated hydrogels (n=3). **(D)** Representative maximum projection fluorescence images of NPCs encapsulated in benzaldehyde crosslinked sulfated hydrogels after 7 days of culture stained for neural stem cell markers Nestin (red) and SOX2 (green) and DAPI (blue). Quantification of the percentage of **(E)** Nestin+ cells and **(F)** SOX2+ after 7 days in culture (n = 5-6). **(G)** Quantification of the percentage of SOX2+ cells that are in clusters vs. single cells after 7 days in culture (n = 5-6). Statistical analyses performed as lognormal unpaired t-test (B), and two-way ANOVA with Bonferroni multiple comparisons test (C and E to G). *$p < 0.05$, ** $p < 0.01$. Data plotted as mean ± standard deviation. Confidence intervals and p-values are reported in Supplemental Table S17 (C) and Supplemental Table S (E to G).

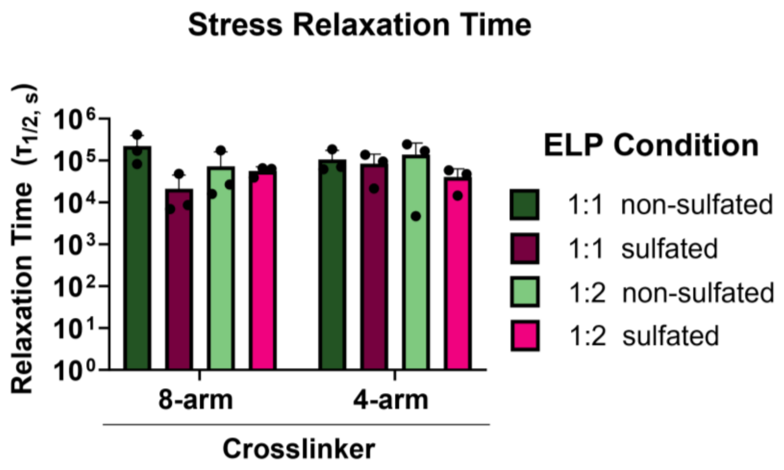


**Supplemental Figure S9.** Stress relaxation half-times ($\tau_{1/2}$) of sulfated versus non-sulfated benzaldehyde crosslinked hydrogels (n = 3). Statistical analyses performed as lognormal unpaired t-tests. Data plotted as mean ± standard deviation.

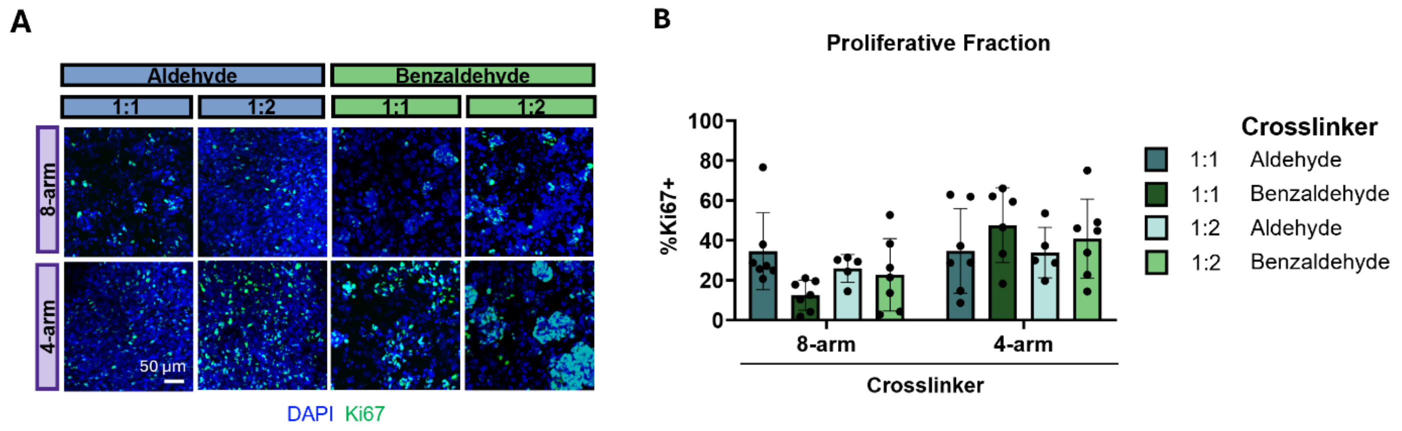


**Supplemental Figure S10. (A)** Representative maximum projection fluorescence images of NPCs encapsulated in sulfated aliphatic aldehyde and benzaldehyde crosslinked hydrogels after 7 days of culture stained for the proliferation marker Ki67 (green) and DAPI (blue). **(B)** Quantification of the percentage of Ki67+ cells after 7 days in culture (n = 3 or 4). Statistical analyses performed as two-way ANOVA with Bonferroni multiple comparisons test (B). Data plotted as mean ± standard deviation. Confidence intervals and p-values are reported in Supplemental Table S8 (B).

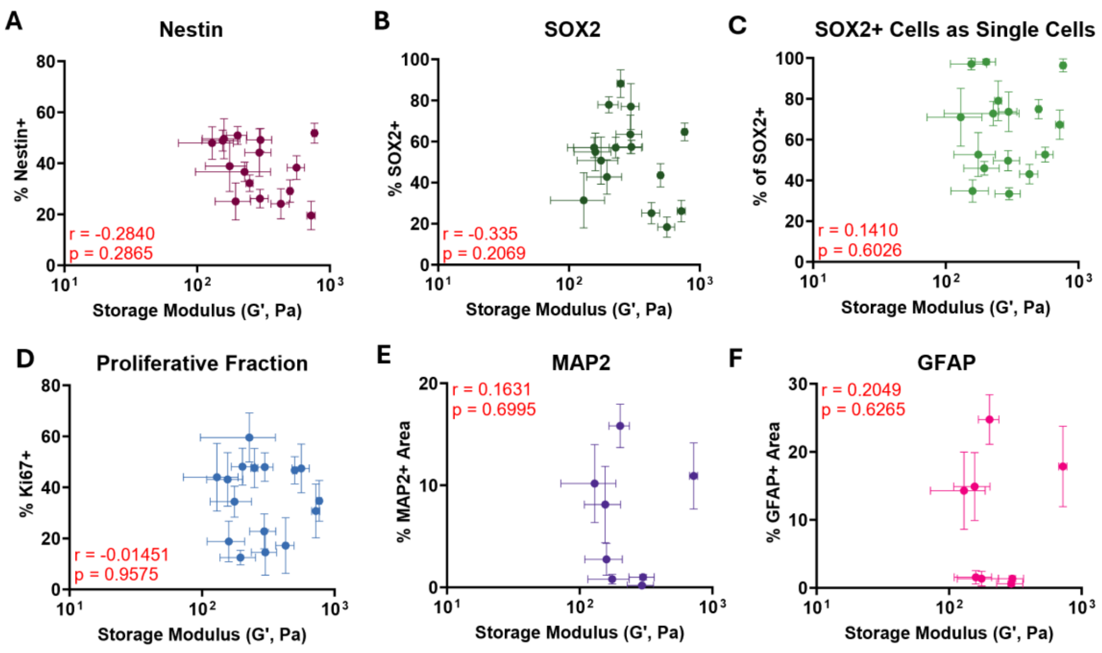


**Supplemental Figure S11.** The percentages of **(A)** Nestin+ cells, **(B)** SOX2+ cells, **(C)** Sox+ cells as single cells vs. clusters, **(D)** Ki67+ cells, **(E)** GFAP+ cells, and **(F)** MAP2+ cells are not correlated with hydrogel storage modulus. Statistical analyses performed as Pearson correlation tests (A to F). Data plotted as mean ± standard error.

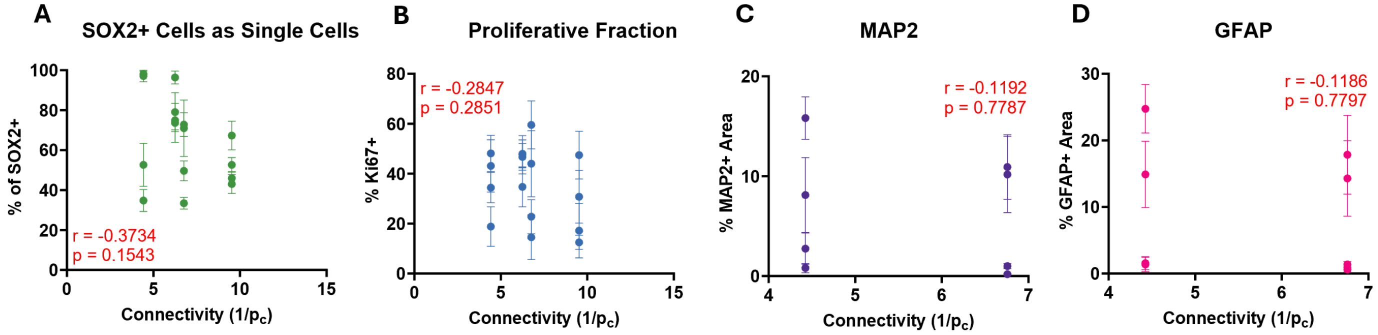


**Supplemental Figure S12.** The percentages of **(A)** SOX2+ cells as single cells **(B)** Ki67+ cells **(C)** MAP2+ cells **(D)** GFAP+ cells are not correlated with hydrogel connectivity. Statistical analyses performed as Pearson correlation tests (A to F). Data plotted as mean ± standard error.

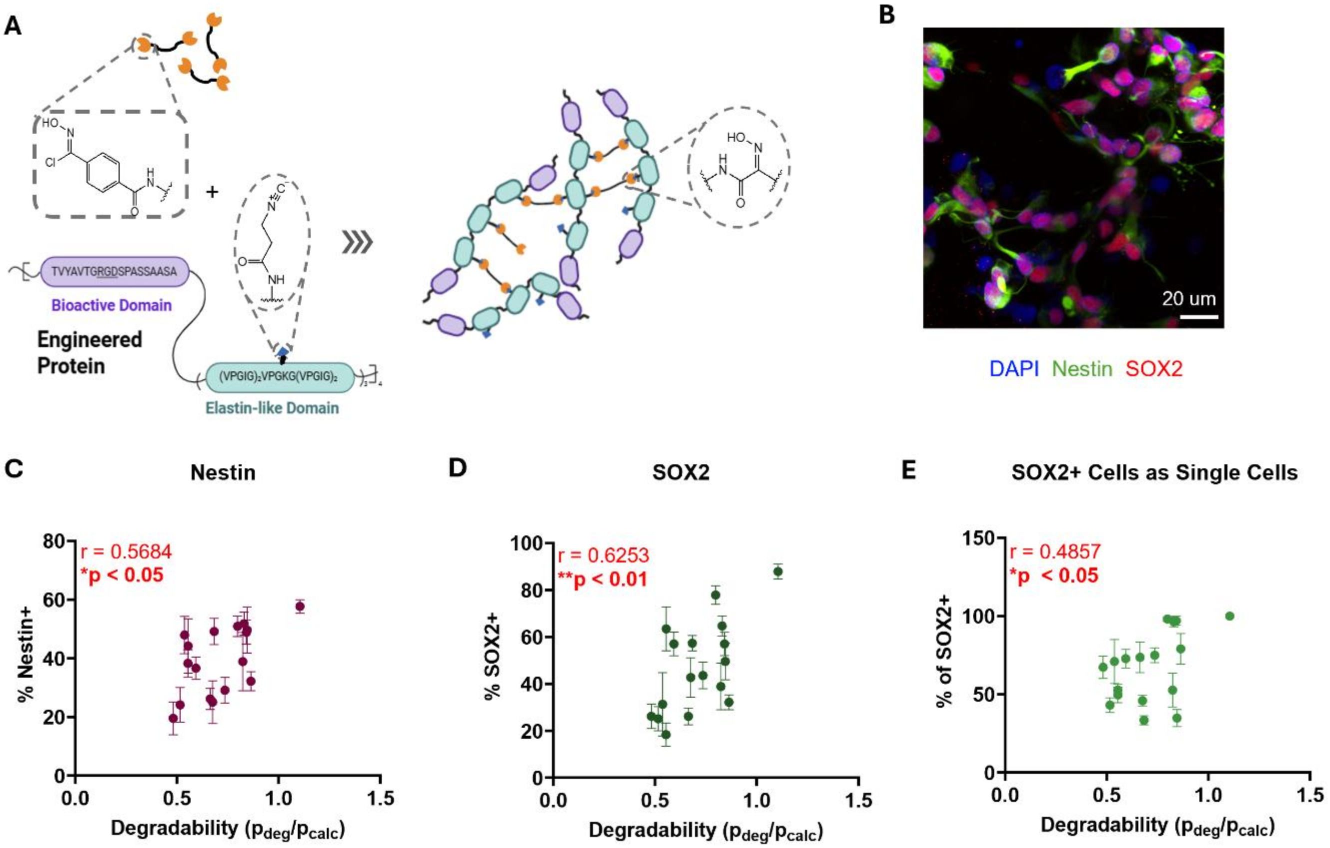


**Supplemental Figure S13. (A)** Schematic of RGD-containing, isonitrile-functionalized ELPs cross–linked with PEG-bis-ChO. **(B)** Representative maximum projection fluorescence image of hNPCs encapsulated in isonitrile-functionalized ELPs cross-linked with PEG-bis-ChO after 7 days of culture stained for neural stem cell markers Nestin (green) and SOX2 (red) and DAPI (blue). The percentages of **(C)** Nestin+ and **(D)** SOX2+ and **(E)** SOX2+ cells that are single cells vs. in clusters and cells increases with increasing degradability. Statistical analyses performed as Pearson correlation tests (C to E). *$p < 0.5$, ** $p < 0.01$.

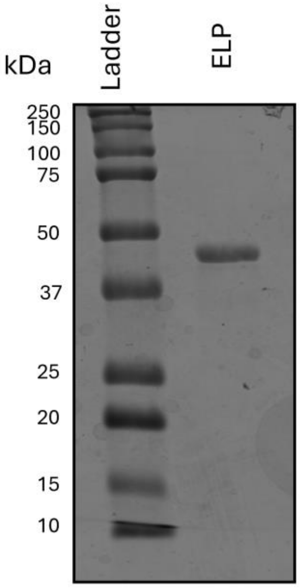


**Supplemental Figure S14.** Representative SDS PAGE gel of ELP with a molecular weight of ~38 kDa post-expression and purification.

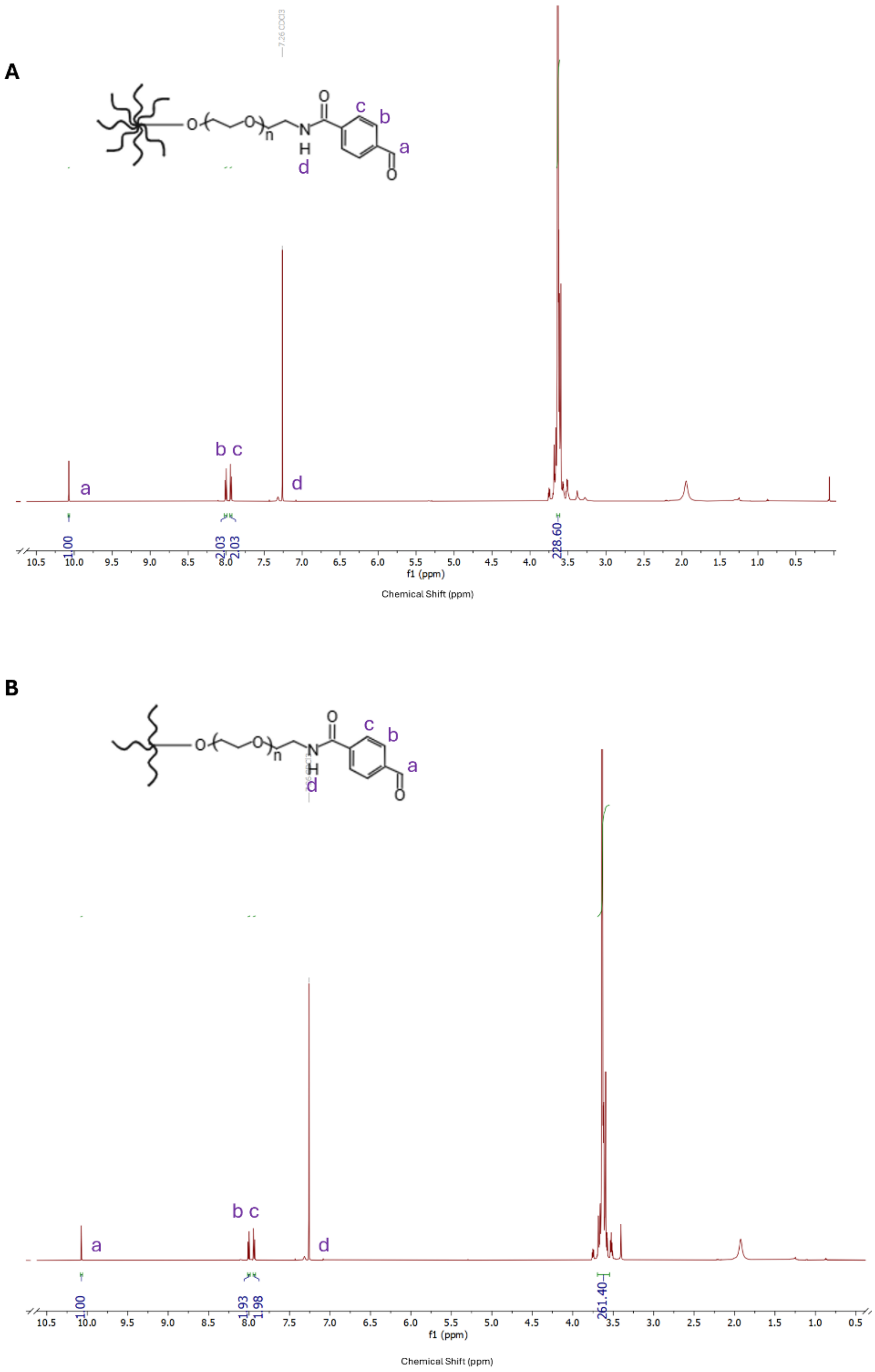


**Supplemental Figure S15. (A)** $^1$H NMR of 20 kDa 8-arm PEG-benzaldehyde in $CDCl_3$ and **(B)** $^1$H NMR of 10 kDa 4-arm PEG-benzaldehyde in $CDCl_3$.

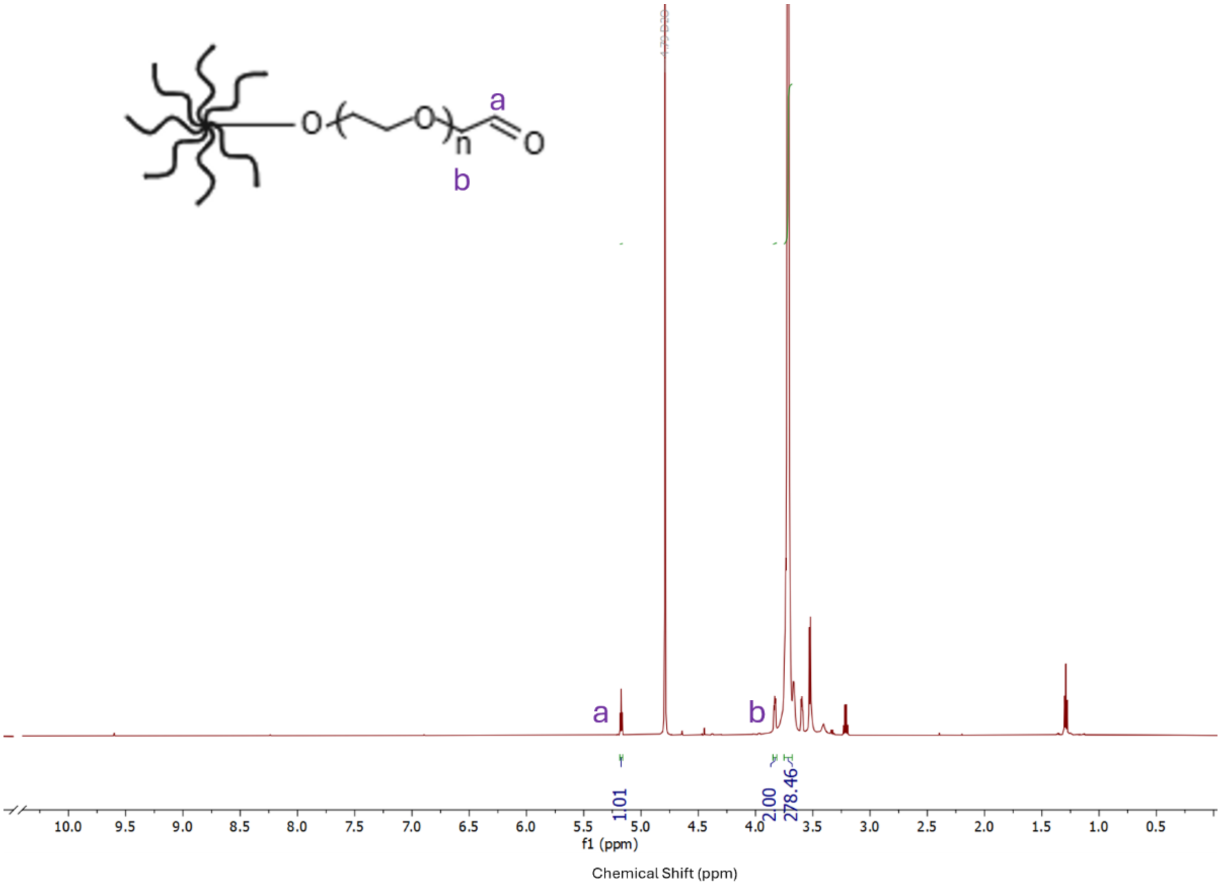


**Supplemental Figure S16.** $^{1}H$ NMR of 20 kDa 8-arm PEG-aldehyde in $D_2O$.

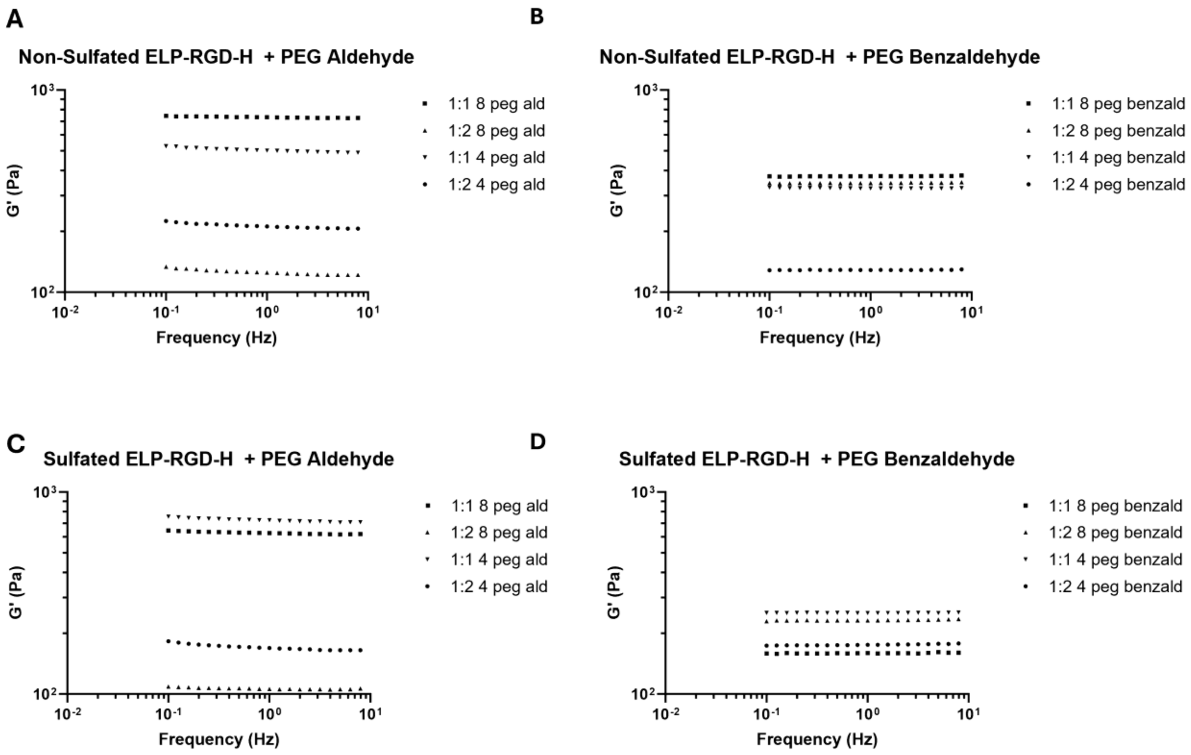


**Supplemental Figure S17. (A-D)** Representative frequency sweeps performed at a constant 3% strain and oscillatory frequency ranging from 0.1Hz to 10Hz at 37°C.

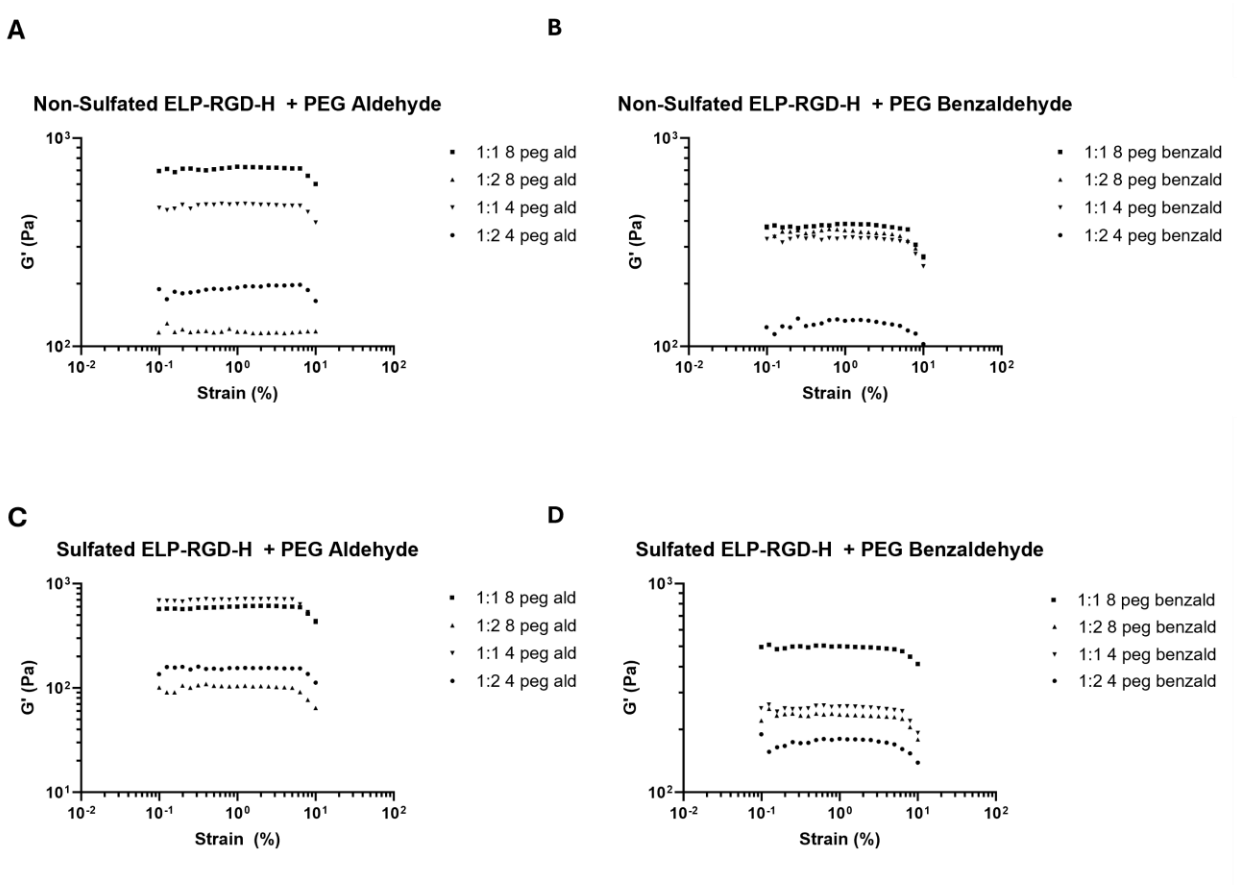


**Supplemental Figure S18. (A to D)** Representative strain sweeps performed at constant oscillatory frequency of 1Hz and initial and final strains of 0.1% and 10%, respectively, at 37℃.

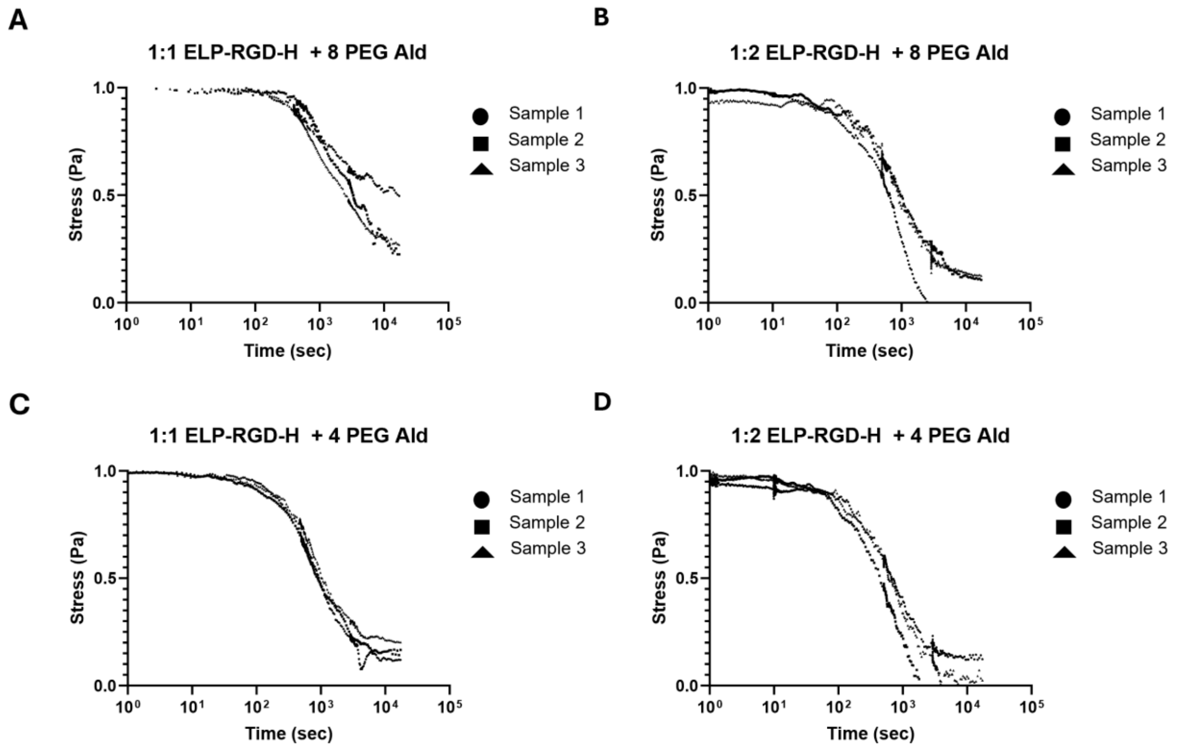


**Supplemental Figure S19. (A to D)** Representative stress relaxation tests of Non-Sulfated ELP and PEG Aldehyde crosslinked gels completed under a constant strain of 10% at 37°C. Stress relaxation values for PEG-Aldehyde crosslinked hydrogels were taken from measured stress relaxation $\tau_{1/2}$ values.

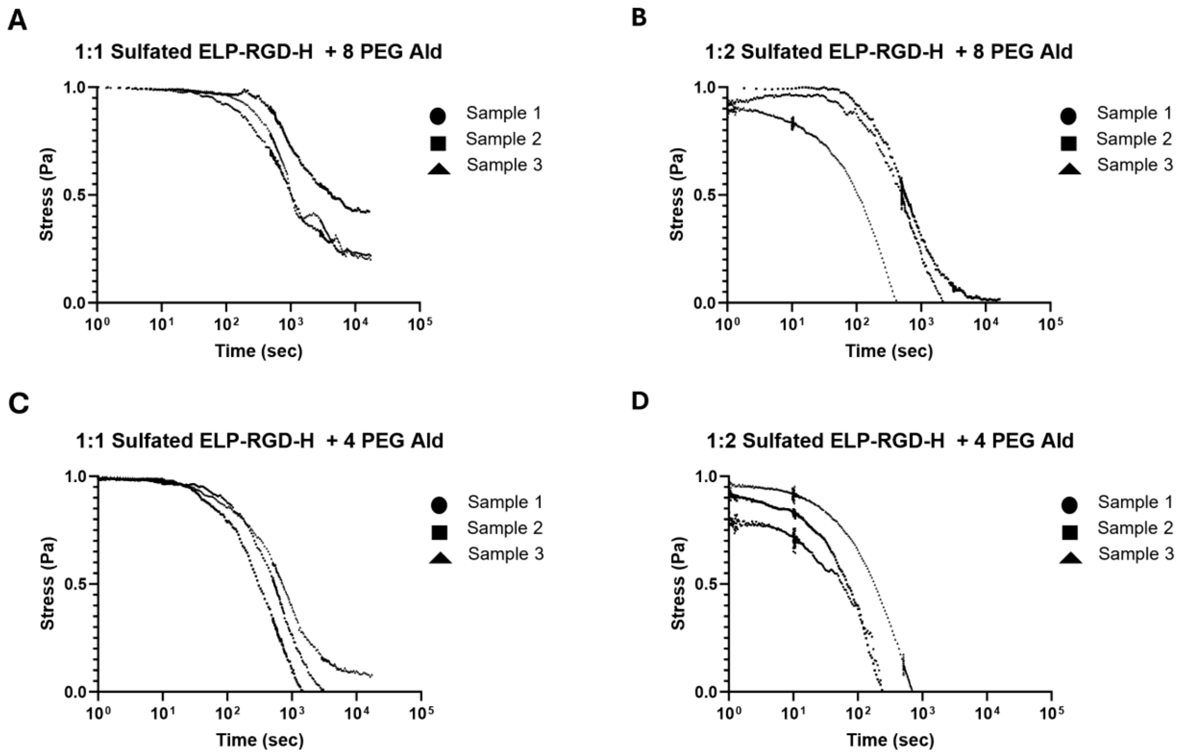


**Supplemental Figure S20. (A to D)** Representative stress relaxation tests of Sulfated ELP and PEG Aldehyde crosslinked gels completed under a constant strain of 10% at 37°C. Stress relaxation values for PEG-Aldehyde crosslinked hydrogels were taken from measured stress relaxation $\tau_{1/2}$ values.

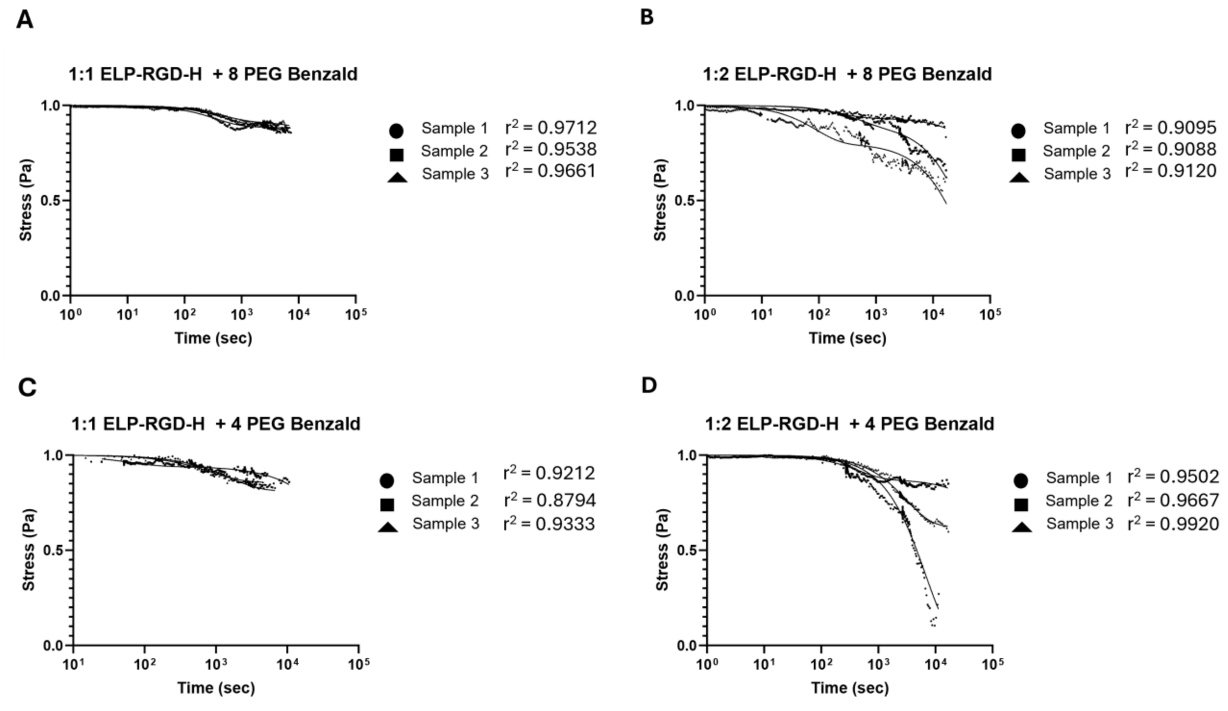


**Supplemental Figure S21. (A to D)** Representative stress relaxation tests of Non-Sulfated ELP and PEG Benzaldehyde crosslinked gels completed under a constant strain of 10% at 37°C. Stress relaxation values for PEG-Benzaldehyde crosslinked hydrogels were estimated based on fitting to a double Maxwell model. Fits and $r^2$ values are plotted in A through D.

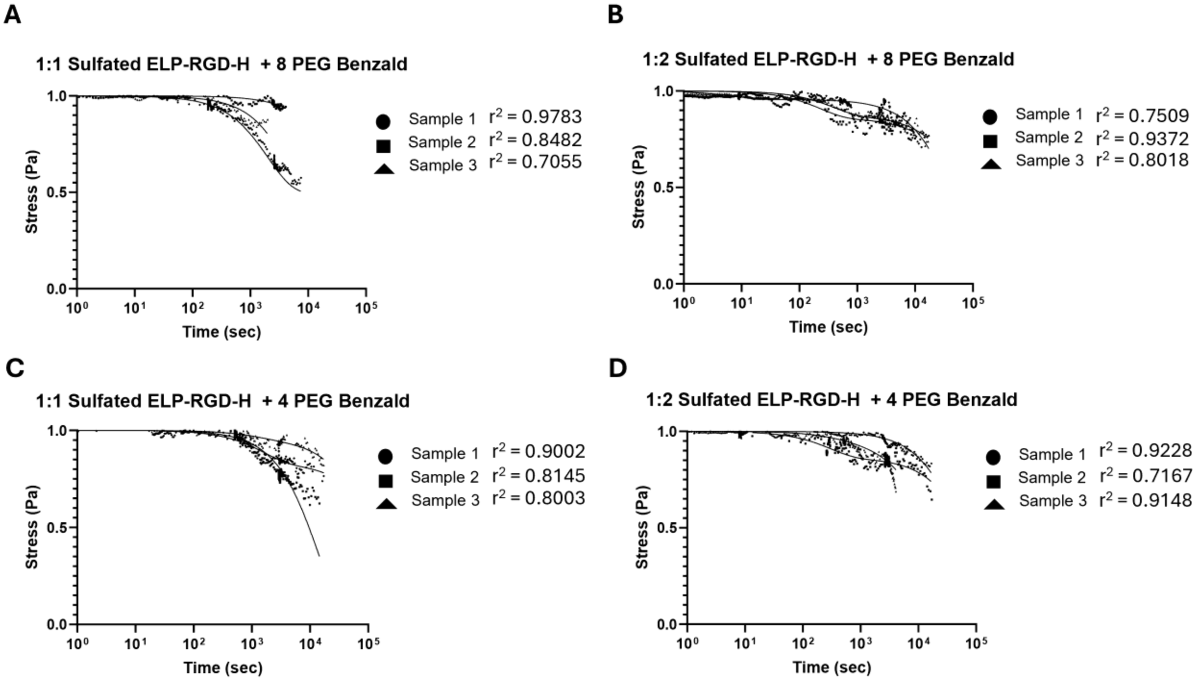


**Supplemental Figure S22. (A to D)** Representative stress relaxation tests of Sulfated ELP and PEG Benzaldehyde crosslinked gels completed under a constant strain of 10% at 37°C. Stress relaxation values for PEG-Benzaldehyde crosslinked hydrogels were estimated based on fitting to a double Maxwell model. Fits and $r^2$ values are plotted in A through D.

**Supplemental Table 1. Stemness Markers Statistical Analyses**

| Figure | Statistical Test | 1:1 – 1:2 8-arm 95% Confidence Interval of Diff | 1:1-1:2 4-arm 95% Confidence Interval of Diff | 8-arm 4-arm 1:1 95% Confidence Interval of Diff | 8-arm 4-arm 1:2 95% Confidence Interval of Diff |
|---|---|---|---|---|---|
| Fig.1 F | Two-way ANOVA | -32.15 to -2.094 *p = 0.0237 | -37.94 to -5.628 **p = 0.0073 | -24.65 to 5.413 p = 0.2783 | -30.43 to 1.880 p = 0.0904 |
| Fig.1 G | Two-way ANOVA | -33.89 to -0.9305 **p = 0.003 | -38.61 to -3.170 **p = 0.002 | -47.31 to -14.35 *p = 0.0371 | -52.03 to -16.59 *p = 0.0190 |
| Fig.1 H | Two-way ANOVA | -24.06 to 13.06 p = 0.9714 | -43.22 to -3.316 *p = 0.0204 | -26.15 to 10.97 p = 0.6758 | -45.31 to -5.410 *p = 0.0113 |
| Fig.2 E | Two-way ANOVA | -44.50 to -5.487 *p = 0.0112 | -42.96 to -3.945 *p = 0.0172 | -21.51 to 17.50 p > 0.9999 | -19.97 to 19.04 p > 0.9999 |
| Fig.2 F | Two-way ANOVA | -59.10 to -5.303 *p = 0.0177 | -4.826 to 48.98 p = 0.1212 | -78.75 to -24.94 ***p = 0.0003 | -24.47 to 29.33 p > 0.9999 |
| Fig.2 G | Two-way ANOVA | -6.189 to 25.52 p = 0.3104 | -57.20 to -25.49 ****p < 0.0001 | -7.549 to 24.16 p = 0.4378 | -58.56 to -26.85 ****p < 0.0001 |
| Fig.3 G | Two-way ANOVA | -26.24 to 6.925 p = 0.3431 | -13.63 to 19.54 p > 0.9999 | -29.35 to 2.280 p = 0.1017 | -18.24 to 16.41 p > 0.9999 |
| Fig.3 H | Two-way ANOVA | -38.70 to 12.68 p = 0.4630 | -18.01 to 33.37 p = 0.9485 | -70.83 to -21.84 ***p = 0.0004 | -52.48 to 1.191 p = 0.0625 |
| Fig.3 I | Two-way ANOVA | -42.67 to 6.063 p = 0.1657 | -25.08 to 23.65 p > 0.9999 | -66.94 to -20.48 ***p = 0.0004 | -51.57 to -0.6732 *p = 0.0437 |
| S5. E | Two-way ANOVA | -46.08 to 7.802 p = 0.2012 | -33.64 to 20.24 p > 0.9999 | -34.05 to 19.83 p > 0.9999 | -21.61 to 32.37 p > 0.9999 |
| S5. F | Two-way ANOVA | -52.07 to 10.64 p = 0.2502 | 6.075 to 68.79 *p = 0.0180 | -76.79 to -14.08 **p = 0.0044 | -18.65 to 44.07 p = 0.6754 |

| | | | | | |
|---|---|---|---|---|---|
| S5. G | Two-way ANOVA | -30.64 to 23.27<br>p > 0.9999 | -0.5332 to 53.37<br>p = 0.0553 | -60.07 to -6.170<br>*p = 0.0149 | -29.97 to 23.93<br>p > 0.9999 |

**Supplemental Table 2. Ki67 Non-Sulfated Aldehyde vs Benzaldehyde Two-way ANOVA with Bonferroni's multiple comparisons test Statistical Analyses**

| Comparison | 95% Confidence Interval of Diff | |
|---|---|---|
| 8-arm 1:1 Aldehyde vs 1:1 Benzaldehyde | 1.290 to 59.83 | *p = 0.0364 |
| 8-arm 1:1 Aldehyde vs 1:2 Aldehyde | -54.47 to 7.143 | p = 0.2346 |
| 8-arm 1:1 Aldehyde vs 1:2 Benzaldehyde | -6.761 to 49.48 | p = 0.2472 |
| 8-arm 1:1 Benzaldehyde vs 1:2 Aldehyde | -86.08 to -22.36 | ***p = 0.0002 |
| 8-arm 1:1 Benzaldehyde vs 1:2 Benzaldehyde | -38.47 to 20.07 | p > 0.9999 |
| 8-arm 1:2 Aldehyde vs 1:2 Benzaldehyde | 14.22 to 75.83 | **p = 0.0014 |
| 4-arm 1:1 Aldehyde vs 1:1 Benzaldehyde | -47.61 to 13.14 | p = 0.7373 |
| 4-arm 1:1 Aldehyde vs 1:2 Aldehyde | -23.64 to 40.07 | p > 0.9999 |
| 4-arm 1:1 Aldehyde vs 1:2 Benzaldehyde | 9.483 to 73.19 | **p = 0.0052 |
| 4-arm 1:1 Benzaldehyde vs 1:2 Aldehyde | -6.410 to 57.30 | p = 0.1937 |
| 4-arm 1:1 Benzaldehyde vs 1:2 Benzaldehyde | 26.71 to 90.43 | ****p < 0.0001 |
| 4-arm 1:2 Aldehyde vs 1:2 Benzaldehyde | -0.1476 to 66.40 | p = 0.0516 |

**Supplemental Table 3. MAP2 Two-way ANOVA with Bonferroni's multiple comparisons test Statistical Analyses**

| Comparison | 95% Confidence Interval of Diff |
|---|---|
| 8-arm Non-Sulfated Aldehyde vs 1:2 Sulfated Aldehyde | -9.703 to 11.20 p > 0.9999 |
| 8-arm Non-Sulfated Aldehyde vs 1:2 Non-Sulfated Benzaldehyde | -1.037 to 20.89 p = 0.0958 |
| 8-arm Non-Sulfated Aldehyde vs 1:2 Sulfated Benzaldehyde | -0.2285 to 21.70 p = 0.0578 |
| 8-arm Sulfated Aldehyde vs Non-Sulfated Benzaldehyde | -1.788 to 20.14 p = 0.1501 |
| 8-arm Sulfated Aldehyde vs Sulfated Benzaldehyde | -0.9790 to 20.95 p = 0.0924 |
| 8-arm Non-Sulfated Benzaldehyde vs Sulfated Benzaldehyde | -10.64 to 12.26 p > 0.9999 |
| 4-arm Non-Sulfated Aldehyde vs 1:2 Sulfated Aldehyde | -10.52 to 12.38 p > 0.9999 |
| 4-arm Non-Sulfated Aldehyde vs 1:2 Non-Sulfated Benzaldehyde | 2.105 to 24.03 *p = 0.0123 |
| 4-arm Non-Sulfated Aldehyde vs 1:2 Sulfated Benzaldehyde | 2.873 to 27.17 **p = 0.0087 |
| 4-arm Sulfated Aldehyde vs Non-Sulfated Benzaldehyde | 1.175 to 23.10 *p = 0.0231 |
| 4-arm Sulfated Aldehyde vs Sulfated Benzaldehyde | 1.943 to 26.24 *p = 0.0156 |
| 4-arm Non-Sulfated Benzaldehyde vs Sulfated Benzaldehyde | -9.738 to 13.64 p > 0.9999 |

**Supplemental Table 4. GFAP Two-way ANOVA with Bonferroni's multiple comparisons test Statistical Analyses**

| Comparison | 95% Confidence Interval of Diff | |
|---|---|---|
| 8-arm Non-Sulfated Aldehyde vs 1:2 Sulfated Aldehyde | -11.19 to 18.30 | p > 0.9999 |
| 8-arm Non-Sulfated Aldehyde vs 1:2 Non-Sulfated Benzaldehyde | 1.881 to 32.81 | *p = 0.0208 |
| 8-arm Non-Sulfated Aldehyde vs 1:2 Sulfated Benzaldehyde | 1.770 to 32.70 | *p = 0.0219 |
| 8-arm Sulfated Aldehyde vs Non-Sulfated Benzaldehyde | -1.676 to 29.25 | p = 0.1050 |
| 8-arm Sulfated Aldehyde vs Sulfated Benzaldehyde | -1.788 to 29.14 | p = 0.1101 |
| 8-arm Non-Sulfated Benzaldehyde vs Sulfated Benzaldehyde | -16.26 to 16.04 | p > 0.9999 |
| 4-arm Non-Sulfated Aldehyde vs 1:2 Sulfated Aldehyde | -6.299 to 26.01 | p = 0.5793 |
| 4-arm Non-Sulfated Aldehyde vs 1:2 Non-Sulfated Benzaldehyde | 7.728 to 38.66 | **p = 0.0011 |
| 4-arm Non-Sulfated Aldehyde vs 1:2 Sulfated Benzaldehyde | 6.265 to 40.53 | **p = 0.0032 |
| 4-arm Sulfated Aldehyde vs Non-Sulfated Benzaldehyde | -2.126 to 28.80 | p = 0.1271 |
| 4-arm Sulfated Aldehyde vs Sulfated Benzaldehyde | -3.589 to 30.68 | p = 0.2016 |
| 4-arm Non-Sulfated Benzaldehyde vs Sulfated Benzaldehyde | -16.28 to 16.69 | p > 0.9999 |

**Supplemental Table 5. Ki67 Sulfated Aldehyde vs Benzaldehyde Two-way ANOVA with Bonferroni's multiple comparisons test Statistical Analyses**

| Comparison | 95% Confidence Interval of Diff |
|---|---|
| 8-arm 1:1 Aldehyde vs 1:1 Benzaldehyde | -2.845 to 46.93 p = 0.1107 |
| 8-arm 1:1 Aldehyde vs 1:2 Aldehyde | -18.68 to 35.84 p > 0.9999 |
| 8-arm 1:1 Aldehyde vs 1:2 Benzaldehyde | -13.11 to 36.66 p > 0.9999 |
| 8-arm 1:1 Benzaldehyde vs 1:2 Aldehyde | -40.72 to 13.80 p > 0.9999 |
| 8-arm 1:1 Benzaldehyde vs 1:2 Benzaldehyde | -35.16 to 14.62 p > 0.9999 |
| 8-arm 1:2 Aldehyde vs 1:2 Benzaldehyde | -24.07 to 30.45 p > 0.9999 |
| 4-arm 1:1 Aldehyde vs 1:1 Benzaldehyde | -38.78 to 13.02 p > 0.9999 |
| 4-arm 1:1 Aldehyde vs 1:2 Aldehyde | -26.41 to 28.11 p > 0.9999 |
| 4-arm 1:1 Aldehyde vs 1:2 Benzaldehyde | -31.03 to 18.74 p > 0.9999 |
| 4-arm 1:1 Benzaldehyde vs 1:2 Aldehyde | -14.46 to 41.92 p > 0.9999 |
| 4-arm 1:1 Benzaldehyde vs 1:2 Benzaldehyde | -19.16 to 32.64 p > 0.9999 |
| 4-arm 1:2 Aldehyde vs 1:2 Benzaldehyde | -34.25 to 20.27 p > 0.9999 |

**Supplemental Table 6. Nestin Multiple Linear Regression Statistics**

| Parameter Estimates | Variable | Estimate | 95% CI (profile likelihood) | \|t\| | P value | P value summary |
|---|---|---|---|---|---|---|
| β0 | Intercept | 72.13 | 48.59 to 95.68 | 6.676 | <0.0001 | **** |
| β1 | Stiffness | -0.001403 | -0.03060 to 0.02779 | 0.1047 | 0.9184 | ns |
| β2 | Stress Relaxation (log $\tau_{1/2}$) | -2.663 | -7.403 to 2.077 | 1.224 | 0.2444 | ns |
| β3 | Connectivity ($1/p_c$) | -3.416 | -6.589 to -0.2434 | 2.346 | 0.0370 | * |

**Supplemental Table 7. Ki67 Multiple Linear Regression Statistics**

| Parameter Estimates | Variable | Estimate | 95% CI (profile likelihood) | \|t\| | P value | P value summary |
|---|---|---|---|---|---|---|
| β0 | Intercept | 73.08 | 38.61 to 107.6 | 4.619 | 0.0006 | *** |
| β1 | Stiffness | 0.001049 | -0.04170 to 0.04380 | 0.05345 | 0.9583 | ns |
| β2 | Stress Relaxation (log $\tau_{1/2}$) | -7.221 | -14.16 to -0.2800 | 2.267 | 0.0427 | * |
| β3 | Connectivity ($1/p_c$) | -1.443 | -6.089 to 3.202 | 0.6769 | 0.5113 | ns |

**Supplemental Table 8. SOX2 Multiple Linear Regression Statistics**

| Parameter Estimates | Variable | Estimate | 95% CI (profile likelihood) | \|t\| | P value | P value summary |
|---|---|---|---|---|---|---|
| β0 | Intercept | 84.96 | 42.08 to 127.8 | 4.317 | 0.0010 | ** |
| β1 | Stiffness | 0.005903 | -0.04728 to 0.05909 | 0.2418 | 0.8130 | ns |
| β2 | Stress Relaxation (log $\tau_{1/2}$) | 4.643 | -3.991 to 13.28 | 1.172 | 0.2641 | ns |
| β3 | Connectivity ($1/p_c$) | -7.834 | -13.61 to -2.055 | 2.954 | 0.0121 | * |

**Supplemental Table 9. SOX2+ Single Cells Multiple Linear Regression Statistics**

| Parameter Estimates | Variable | Estimate | 95% CI (profile likelihood) | \|t\| | P value | P value summary |
|---|---|---|---|---|---|---|
| β0 | Intercept | 132.1 | 93.44 to 170.8 | 7.440 | <0.0001 | **** |
| β1 | Stiffness | 0.02716 | -0.02083 to 0.07516 | 1.233 | 0.2411 | ns |
| β2 | Stress Relaxation (log $\tau_{1/2}$) | -12.09 | -19.88 to -4.302 | 3.382 | 0.0054 | ** |
| β3 | Connectivity ($1/p_c$) | -4.313 | -9.528 to 0.9019 | 1.802 | 0.0967 | ns |

**Supplemental Table 10. Nestin RGD vs RDG Two-way ANOVA with Bonferroni's multiple comparisons test Statistical Analyses**

| Comparison | 95% Confidence Interval of Diff |
|---|---|
| 1:2 RGD 8-arm vs 1:2 RDG 8-arm Aldehyde | -39.62 to 1.073 p = 0.0723 |
| 1:2 RGD 8-arm vs 1:2 RGD 4-arm Aldehyde | -33.61 to 5.055 p = 0.2797 |
| 1:2 RGD 8-arm vs 1:2 RDG 4-arm Aldehyde | -44.60 to -3.906 *p = 0.0121 |
| 1:2 RDG 8-arm vs 1:2 RGD 4-arm Aldehyde | -16.04 to 26.04 p > 0.9999 |
| 1:2 RDG 8-arm vs 1:2 RDG 4-arm Aldehyde | -26.96 to 17.00 p > 0.9999 |
| 1:2 RGD 4-arm vs 1:2 RDG 4-arm Aldehyde | -31.02 to 11.06 p > 0.9999 |
| 1:2 RGD 8-arm vs 1:2 RDG 8-arm Benzaldehyde | -5.358 to 36.72 p = 0.2684 |
| 1:2 RGD 8-arm vs 1:2 RGD 4-arm Benzaldehyde | -26.33 to 13.79 p > 0.9999 |
| 1:2 RGD 8-arm vs 1:2 RDG 4-arm Benzaldehyde | -3.313 to 38.77 p = 0.1457 |
| 1:2 RDG 8-arm vs 1:2 RGD 4-arm Benzaldehyde | -42.99 to -0.9107 *p =0.0367 |
| 1:2 RDG 8-arm vs 1:2 RDG 4-arm Benzaldehyde | -19.93 to 24.02 p > 0.9999 |
| 1:2 RGD 4-arm vs 1:2 RDG 4-arm Benzaldehyde | 2.956 to 45.04 p = 0.0179 |

**Supplemental Table 11. SOX2 RGD vs RDG Two-way ANOVA with Bonferroni's multiple comparisons test Statistical Analyses**

| Comparison | 95% Confidence Interval of Diff |
|---|---|
| 1:2 RGD 8-arm vs 1:2 RDG 8-arm Aldehyde | -48.86 to -8.638 **p = 0.0018 |
| 1:2 RGD 8-arm vs 1:2 RGD 4-arm Aldehyde | -33.39 to 4.833 p = 0.2655 |
| 1:2 RGD 8-arm vs 1:2 RDG 4-arm Aldehyde | -52.50 to -11.97 ***p = 0.0005 |
| 1:2 RDG 8-arm vs 1:2 RGD 4-arm Aldehyde | -6.325 to 35.27 p = 0.3603 |
| 1:2 RDG 8-arm vs 1:2 RDG 4-arm Aldehyde | -25.06 to 18.39 p > 0.9999 |
| 1:2 RGD 4-arm vs 1:2 RDG 4-arm Aldehyde | -38.61 to 2.988 p = 0.1332 |
| 1:2 RGD 8-arm vs 1:2 RDG 8-arm Benzaldehyde | 5.288 to 46.89 **p = 0.0075 |
| 1:2 RGD 8-arm vs 1:2 RGD 4-arm Benzaldehyde | -37.96 to 1.701 p = 0.0906 |
| 1:2 RGD 8-arm vs 1:2 RDG 4-arm Benzaldehyde | 5.372 to 46.97 **p = 0.0072 |
| 1:2 RDG 8-arm vs 1:2 RGD 4-arm Benzaldehyde | -65.02 to -23.42 ****p < 0.0001 |
| 1:2 RDG 8-arm vs 1:2 RDG 4-arm Benzaldehyde | -21.64 to 21.81 p > 0.9999 |
| 1:2 RGD 4-arm vs 1:2 RDG 4-arm Benzaldehyde | 23.50 to 65.10 ****p < 0.0001 |

**Supplemental Table 12. SOX2 Single Cells RGD vs RDG Two-way ANOVA with Bonferroni's multiple comparisons test Statistical Analyses**

| Comparison | 95% Confidence Interval of Diff |
|---|---|
| 1:2 RGD 8-arm vs 1:2 RDG 8-arm Aldehyde | 52.43 to 83.47 ****p < 0.0001 |
| 1:2 RGD 8-arm vs 1:2 RGD 4-arm Aldehyde | -40.11 to -10.61 ***p = 0.0002 |
| 1:2 RGD 8-arm vs 1:2 RDG 4-arm Aldehyde | 52.43 to 83.47 ****p < 0.0001 |
| 1:2 RDG 8-arm vs 1:2 RGD 4-arm Aldehyde | -109.4 to -77.26 ****p < 0.0001 |
| 1:2 RDG 8-arm vs 1:2 RDG 4-arm Aldehyde | -16.77 to 16.76 p > 0.9999 |
| 1:2 RGD 4-arm vs 1:2 RDG 4-arm Aldehyde | 77.26 to 109.4 ****p < 0.0001 |
| 1:2 RGD 8-arm vs 1:2 RDG 8-arm Benzaldehyde | -6.237 to 25.87 p = 0.5800 |
| 1:2 RGD 8-arm vs 1:2 RGD 4-arm Benzaldehyde | -16.66 to 13.94 p > 0.9999 |
| 1:2 RGD 8-arm vs 1:2 RDG 4-arm Benzaldehyde | -1.480 to 30.62 p = 0.0946 |
| 1:2 RDG 8-arm vs 1:2 RGD 4-arm Benzaldehyde | -27.23 to 4.877 p = 0.3597 |
| 1:2 RDG 8-arm vs 1:2 RDG 4-arm Benzaldehyde | -12.01 to 21.52 p > 0.9999 |
| 1:2 RGD 4-arm vs 1:2 RDG 4-arm Benzaldehyde | -0.1198 to 31.98 p = 0.0527 |

**Supplemental Table 13. N-Cadherin Inhibition β-Catenin Activity Two-way ANOVA with Bonferroni's multiple comparisons test Statistical Analyses**

| 8-arm vs 4-arm Aldehyde 95% Confidence Interval of Diff | 8-arm vs 4-arm Benzaldehyde 95% Confidence Interval of Diff | Aldehyde vs Benzaldehyde 8-arm 95% Confidence Interval of Diff | Aldehyde vs Benzaldehyde 4-arm 95% Confidence Interval of Diff |
|---|---|---|---|
| -0.2178 to -0.05185<br>**p = 0.0023 | -0.1277 to 0.02381<br>p = 0.2146 | 0.01761 to 0.1691<br>*p = 0.0159 | 0.09327 to 0.2592<br>***p = 0.0002 |

**Supplemental Table 14. N-Cadherin Inhibition Stemness Maintenance Two-way ANOVA with Bonferroni's multiple comparisons test Statistical Analyses**

| Control vs cHAV Nestin 95% Confidence Interval of Diff | Control vs cHAV SOX2 95% Confidence Interval of Diff | Nestin vs SOX2 Control 95% Confidence Interval of Diff | Nestin vs SOX2 cHAV 95% Confidence Interval of Diff |
|---|---|---|---|
| 41.75 to 59.01<br>****$p < 0.0001$ | 85.51 to 102.8<br>****$p < 0.0001$ | -52.77 to -34.75<br>****$p < 0.0001$ | -8.227 to 8.227<br>$p > 0.9999$ |

**Supplemental Table 15. Theoretical Prediction of Extent of Reaction and Network Degradability**

| Gel Composition | Extents of Reaction (p) | Critical Gelation Points ($p_c$) | Critical Gelation Points After Degradation ($p_{c,deg}$) | Degradability ($p_{c,deg}/p$) |
|---|---|---|---|---|
| 1:1 ELP-H 8 PEG Ald | 0.554333096 | 0.104828 | 0.267261242 | 0.482131 |
| 1:2 ELP-H 8 PEG Ald | 0.636787849 | 0.14825 | 0.377964473 | 0.593549 |
| 1:1 ELP-H 4 PEG Ald | 0.554782573 | 0.160128 | 0.40824829 | 0.735871 |
| 1:2 ELP-H 4 PEG Ald | 0.723175439 | 0.226455 | 0.577350269 | 0.798354 |
| 1:1 ELP-H 8 PEG Benzald | 0.481942558 | 0.104828 | 0.267261242 | 0.55455 |
| 1:2 ELP-H 8 PEG Benzald | 0.70297737 | 0.14825 | 0.377964473 | 0.537662 |
| 1:1 ELP-H 4 PEG Benzald | 0.491812716 | 0.160128 | 0.40824829 | 0.830089 |
| 1:2 ELP-H 4 PEG Benzald | 0.685577248 | 0.226455 | 0.577350269 | 0.842137 |
| 1:1 ELP-CysA-H 8 PEG Ald | 0.517698749 | 0.104828 | 0.267261242 | 0.516249 |
| 1:2 ELP-CysA-H 8 PEG Ald | 0.553179271 | 0.14825 | 0.377964473 | 0.683259 |
| 1:1 ELP-CysA-H 4 PEG Ald | 0.614985823 | 0.160128 | 0.40824829 | 0.663834 |
| 1:2 ELP-CysA-H 4 PEG Ald | 0.682315513 | 0.226455 | 0.577350269 | 0.846163 |
| 1:1 ELP-CysA-H 8 PEG Benzald | 0.395174841 | 0.104828 | 0.267261242 | 0.676311 |
| 1:2 ELP-CysA-H 8 PEG Benzald | 0.680693519 | 0.14825 | 0.377964473 | 0.555264 |
| 1:1 ELP-CysA-H 4 PEG Benzald | 0.472647652 | 0.160128 | 0.40824829 | 0.863748 |
| 1:2 ELP-CysA-H 4 PEG Benzald | 0.700692812 | 0.226455 | 0.577350269 | 0.823971 |
| bis-PEG-NC ELP | 0.63994537 | 0.27735 | 0.707106781 | 1.104949 |

**Supplemental Table 16.** Hydrogel formulation wt% values.

| Gel Composition | ELP wt% | PEG wt% |
|---|---|---|
| 1:1 ELP-H 8 PEG Ald | 3% | 2.76% 8-arm 20kDa |
| 1:2 ELP-H 8 PEG Ald | 3% | 5.52% 8-arm 20kDa |
| 1:1 ELP-H 4 PEG Ald | 3% | 2.76% 4-arm 10kDa |
| 1:2 ELP-H 4 PEG Ald | 3% | 5.52% 4-arm 10kDa |
| 1:1 ELP-H 8 PEG Benzald | 3% | 3.41% 8-arm 20kDa |
| 1:2 ELP-H 8 PEG Benzald | 3% | 6.82% 8-arm 20kDa |
| 1:1 ELP-H 4 PEG Benzald | 3% | 3.80% 4-arm 10kDa |
| 1:2 ELP-H 4 PEG Benzald | 3% | 7.6% 4-arm 10kDa |

**Supplemental Table 17. Storage Modulus Statistical Analyses**

| Figure | Statistical Test | 1:1 – 1:2 8-arm 95% Confidence Interval of Diff | 1:1-1:2 4-arm 95% Confidence Interval of Diff | 8-arm – 4-arm 1:1 95% Confidence Interval of Diff | 8-arm – 4-arm 1:2 95% Confidence Interval of Diff |
|---|---|---|---|---|---|
| S2. | Two-way ANOVA | 212.3 to 779.6<br>**p = 0.0027 | 15.26 to 582.5<br>*p = 0.0398 | -60.70 to 506.6<br>p = 0.1251 | -257.8 to 309.5<br>p > 0.9999 |
| S4. | Two-way ANOVA | -97.16 to 351.9<br>p = 0.3144 | -86.63 to 362.4<br>p = 0.2591 | -94.45 to 354.6<br>p = 0.2992 | -83.92 to 365.1<br>p = 0.2463 |
| S6. | Two-way ANOVA | 206.2 to 656.0<br>**p = 0.0015 | 385.6 to 835.4<br>***p = 0.0001 | -430.7 to 19.10<br>p = 0.0719 | -251.2 to 198.6<br>p > 0.9999 |
| S8. C | Two-way ANOVA | -309.6 to 110.3<br>p = 0.4555 | -136.9 to 283.0<br>p = 0.7333 | -263.6 to 156.4<br>p > 0.9999 | -90.87 to 329.1<br>p = 0.3145 |

**Supplemental Table 18. Primary Antibodies**

| Target | Host Species | Supplier | Catalog Number | Dilution |
|---|---|---|---|---|
| Nestin | Mouse | Millipore Sigma | MAB5326 | 1:600 |
| SOX2 | Rabbit | Cell Signaling | 23064S | 1:400 |
| Ki67 | Rabbit | Cell Signaling | 9129S | 1:400 |
| MAP2 | Rabbit | Cell Signaling | 8707S | 1:400 |
| GFAP | Chicken | AVES Labs | GFAP | 1:300 |
| Non-phospho- $\beta$ -catenin (active S33/S37/T41) | Rat | BioLegend | 631851 | 1:500 |

**Supplemental Table 19. Secondary Antibodies**

| Target | Conjugate | Supplier | Catalog Number | Dilution |
|---|---|---|---|---|
| Goat Anti-Mouse IgG1 | AlexaFluor 647 | Jackson ImmunoResearch | 115-605-205 | 1:600 |
| Goat Anti-Rabbit IgG (H+L) | AlexaFluor 488 | Jackson ImmunoResearch | 111-545-144 | 1:400 |
| Goat Anti-Chicken IgY | AlexaFluor 647 | Jackson ImmunoResearch | 103-605-155 | 1:300 |
| Goat Anti-Rat IgG | AlexaFluor 488 | Jackson Immunoresearch | 112-545-167 | 1:500 |

**Supporting References:**


1 Douglas R. Miller, C. W. M. A New Derivation of Post Gel Properties of Network Polymers. *Macromolecules* **9**, 206-211 (1976).

2 Flory, P. J. Statistical thermodynamics of random networks. *Proceedings of the Royal Society of London. A. Mathematical and Physical Sciences* **351** (1976).

3 Enrique M. Valles, C. W. M. Properties of Networks Formed by End Linking of Poly(dimethylsiloxane). *Macromolecules* **12** (1979).

4 Dietrich Stauffer, A. C. M. A. Vol. 44 (Advances in Polymer Science, 2005).

5 Mithieux, S. M. & Weiss, A. S. Elastin. *Adv Protein Chem* **70**, 437-461, doi:10.1016/S0065-3233(05)70013-9 (2005).

6 Madl, C. M. *et al.* Maintenance of neural progenitor cell stemness in 3D hydrogels requires matrix remodelling. *Nat Mater* **16**, 1233-1242, doi:10.1038/nmat5020 (2017).

7 Mingjiang Zhong, R. W., Ken Kawamoto, Bradley D. Olsen, and Jeremiah A. Johnson. Quantifying the impact of molecular defects on polymer network elasticity. *Science* **353**, 1264-1268, doi:10.1126/science.aag0184 (2016).
